# Camrelizumab plus famitinib as first-line treatment for locally advanced or metastatic pulmonary sarcomatoid carcinomas (CAPSTONE): a multi-center, single-arm, phase 2 trial

**DOI:** 10.1016/j.eclinm.2026.104285

**Published:** 2026-10-24

**Authors:** Jizong Jiang, Yuxuan Wei, Bin Yang, Xianling Liu, Shundong Cang, Qibin Song, Huijuan Wang, Zheng Jin, Jun Wang, Feng Ye, Fenglin She, Hongyi Kou, Bo Zhu, Qian Chu

**Affiliations:** a Department of Oncology, Tongji Hospital of Tongji Medical College, Huazhong University of Science and Technology, Wuhan, China; b Department of Medical Oncology, Hubei Cancer Hospital, Wuhan, China; c Department of Oncology, The Second Xiangya Hospital, Central South University, Changsha, China; d Department of Medical Oncology, Henan Provincial People's Hospital, Zhengzhou, China; e Cancer Center, Renmin Hospital of Wuhan University, Wuhan, China; f Department of Medical Oncology, Henan Cancer Hospital, Zhengzhou, China; g Institute of Immunological Innovation and Translation, Chongqing Medical University, Chongqing, China; h Department of Oncology, The First Affiliated Hospital of Shandong First Medical University, Jinan, China; i Department of Medical Oncology, The First Affiliated Hospital of Xiamen University, Xiamen, China; j Jiangsu Hengrui Pharmaceuticals Co, Ltd, Shanghai, China; k Institute of Cancer, Xinqiao Hospital, Third Military Medical University, Chongqing, China

**Keywords:** Pulmonary sarcomatoid carcinomas, Rare tumor, Camrelizumab, Famitinib

## Abstract

**Background:**

The combination of immune checkpoint inhibitors and antiangiogenic agents has demonstrated a synergistic antitumor effect in numerous solid tumors, but has rarely been investigated in pulmonary sarcomatoid carcinoma (PSC). We assessed the activity and safety of camrelizumab (a programmed cell death-1 inhibitor) plus famitinib (an antiangiogenic agent) in patients with PSC.

**Methods:**

CAPSTONE is an open-label, single-arm, Simon two-stage, phase 2 trial done at eight sites in China. Eligible patients were aged between 18 and 75 years and had previously untreated stage IIIB–IV pulmonary sarcomatoid carcinoma, an Eastern Cooperative Oncology Group performance status of 0 or 1, and at least one measurable lesion as per Response Evaluation Criteria in Solid Tumors (RECIST) version 1.1. Patients received intravenous camrelizumab 200 mg every 3 weeks and oral famitinib 20 mg once daily until disease progression or intolerable toxicity. The primary endpoint was confirmed objective response rate (ORR) as per RECIST version 1.1 in the full analysis set, which included all patients who received at least one dose of study drug. Safety was assessed in all as-treated patients. This study is registered at ClinicalTrials.gov, NCT04888429, and enrollment is complete.

**Findings:**

Between August 4, 2021 and March 21, 2024, 28 patients were enrolled. With a median follow-up of 18.1 months, the confirmed ORR was 42.9% (95% confidence interval [CI] 24.5–62.8). The disease control rate was 85.7% (95% CI 67.3–96.0). The median duration of response was 16.0 months (95% CI 5.0–29.9); the median progression-free survival was 7.1 months (95% CI 3.5–12.5); and the median overall survival was 18.1 months (95% CI 10.3–28.1). Treatment-related adverse events (TRAEs) of grade 3 or more were observed in 10 (35.7%) patients, the most common being decreased platelet count (four [14.3%]) and decreased neutrophil count (three [10.7%]). Three (10.7%) patients discontinued camrelizumab, and three (10.7%) discontinued famitinib due to TRAEs. Two patients died of unknown cause, which were assessed by the investigator as possibly related to study treatment.

**Interpretation:**

Camrelizumab plus famitinib showed antitumor activity in patients with locally advanced or metastatic PSC. This combination may be a potential first-line option for this patient population, and warrants further investigation in a large-sample trial.

**Funding:**

10.13039/501100018537National Science and Technology Major Project of the Ministry of Science and Technology of China (No. 2024ZD0519700), National Natural Science Foundation of China (62131009, 82472969), Innovation Research Group Program of the Natural Science Foundation of Hubei Province (2026AFA036), Hubei Provincial Health Science and Technology Innovation and Achievement Transformation Project, 10.13039/100018731Jiangsu Hengrui Pharmaceuticals Co., Ltd.

**Research in context**

**Evidence before this study**

We searched PubMed for articles published in English from database inception to May 1, 2026, with the search terms “(“pulmonary sarcomatoid carcinoma” OR “lung sarcomatoid carcinoma”) AND (“immunotherapy” OR “immune checkpoint blockade” OR “immune checkpoint inhibitor” OR “PD-1” OR “PD-L1”)”. We found retrospective studies and case reports showing activity of immunotherapy in pulmonary sarcomatoid carcinoma. The only prospective trial data were derived from two phase 2 trials (i.e. KCSG-LU16-07 and KCSG LU-19-24), which demonstrated the preliminary antitumor activity of durvalumab plus tremelimumab or durvalumab plus doxorubicin and ifosfamide in patients with pulmonary sarcomatoid carcinoma, regardless of prior treatment status. However, there is no prospective evidence on whether the addition of antiangiogenic agent to immunotherapy improves therapeutic efficacy in patients with pulmonary sarcomatoid carcinoma, especially in the first-line setting.

**Added value of this study**

To our knowledge, CAPSTONE is the first multi-center phase 2 trial to report the activity and safety of an immune checkpoint inhibitor in combination with an antiangiogenic agent as first-line therapy in patients with pulmonary sarcomatoid carcinoma. This study showed antitumor activity with camrelizumab plus famitinib in this patient population. Additionally, the toxicity profile observed was consistent with published data.

**Implications of all the available evidence**

Considering the rarity of pulmonary sarcomatoid carcinoma, no phase 3 trials for this disease have been performed to date. Our findings provide preliminary evidence that immunotherapy in combination with antiangiogenic therapy is a potential first-line strategy for patients with pulmonary sarcomatoid carcinoma. Further studies with larger sample sizes are warranted to validate the activity of camrelizumab plus famitinib in this patient population.

## Introduction

Pulmonary sarcomatoid carcinoma (PSC) is a rare subtype of poorly differentiated non-small cell lung cancer (NSCLC), characterized by the presence of both carcinomatous and sarcomatous components.^1^, ^2^ It is estimated to account for about 0.5% of all NSCLC, with a higher prevalence observed in males, smokers, and older patients.^2^, ^3^ In contrast to other subtypes of NSCLC, PSC is more aggressive and is associated with a worse prognosis. Patients with advanced PSC are typically insensitive to conventional radiotherapy and chemotherapy, with a median overall survival (OS) of only 4.3–7.9 months.^4^, ^5^, ^6^ Therefore, it is imperative to pursue the exploration of new treatment strategies for this population.

Immune checkpoint inhibitor (ICI)-based strategies have become a cornerstone of first-line treatment for patients with NSCLC and have improved survival outcomes.^7^, ^8^ Nevertheless, the efficacy of immunotherapy in PSC remains uncertain. Genomic analyses reveal that compared to the conventional subtypes of NSCLC, PSC is characterized by high programmed cell death ligand-1 (PD-L1) expression and the presence of a T-cell inflamed microenvironment.^9^, ^10^, ^11^ Therefore, in theory, immunotherapy is a promising strategy for patients with PSC. Meanwhile, high PD-L1 expression in PSC is accompanied by overexpression of hypoxia-inducible factor-1α (HIF-1α), with this co-expression being more frequently observed than in conventional NSCLC.^12^ HIF-1α upregulation can induce the expression of pro-angiogenic factors such as vascular endothelial growth factor A (VEGF-A), thereby driving tumor angiogenesis.^13^ In addition, platelet-derived growth factor receptor β (PDGFR-β), an important mediator of angiogenesis, has also been reported to be significantly upregulated in PSC.^14^ These provide a rationale for combining ICIs with antiangiogenic therapy in PSC. At present, several case reports and retrospective studies have demonstrated potential activity of this combination for PSC^15^, ^16^, ^17^; however, prospective evidence remains limited.

Camrelizumab, an anti-PD-1 monoclonal antibody, combined with chemotherapy was approved for advanced NSCLC as the first-line setting in China.^18^, ^19^ Famitinib is an oral multi-targeted receptor tyrosine kinase inhibitor that targets stem-cell factor receptor, VEGF receptor-2/3, PDGFR-β, FMS-like tyrosine kinase-3 receptor, and proto-oncogene tyrosine-protein kinase receptor.^20^ Camrelizumab plus famitinib has shown encouraging activity with a manageable toxicity profile in several malignancies.^21^, ^22^, ^23^ In this context, we conducted this single-arm phase 2 trial to assess the activity and safety of camrelizumab combined with famitinib as first-line treatment in patients with locally advanced or metastatic PSC. An exploratory biomarker analysis was also performed to characterize potential genomic features in this rare population.

## Methods

### Study design and patients

CAPSTONE was an open-label, multicentre, single-arm, phase 2 trial done at eight hospitals in China. Eligible patients were aged between 18 and 75 years, with histologically confirmed stage IIIB–IV PSC, who had been deemed unsuitable for surgery and radical chemoradiotherapy, had not previously received systemic therapy, had at least one measurable lesion as per Response Evaluation Criteria in Solid Tumors (RECIST) version 1.1, and had an Eastern Cooperative Oncology Group performance status of 0 or 1. The diagnoses of PSC were established using needle biopsy specimens according to the World Health Organization diagnostic criteria, and all pathological specimens were centrally reviewed by two thoracic pathologists. Key exclusion criteria were the presence of EGFR-sensitizing mutation, ALK or ROS1 alterations, BRAFV600E mutation, or MET exon 14 skipping mutation; active bleeding or a tendency for hemorrhage; uncontrolled hypertension; or active central nervous system metastases. Full eligibility criteria are listed in the Supplementary Protocol.

### Ethics statement

This trial was performed in accordance with the Declarations of Helsinki and Good Clinical Practice guidelines, and was approved by the ethical committee of Tongji Hospital of Tongji Medical College, Huazhong University of Science and Technology (approval number: No. 2021-S037) and all participating sub-sites. All patients provided written informed consent.

### Procedures

Eligible patients received intravenous camrelizumab 200 mg every 3 weeks and oral famitinib 20 mg once daily until disease progression or intolerable toxicity. Dose reduction of camrelizumab was not allowed for the management of adverse events (AEs), whereas dose interruption or permanent discontinuation was permitted. Dose adjustments, including reduction (to 15 mg), interruption and discontinuation, were allowed for famitinib. Once the famitinib dose was reduced, re-escalation was not permitted. The maximum interval for dose interruption was nine weeks for camrelizumab and four weeks for famitinib.

Tumor imaging was conducted using computed tomography or magnetic resonance imaging scans of the chest, upper abdomen, and brain at baseline, every 6 weeks for the first 12 months, and every 12 weeks thereafter. Tumor response was assessed according to RECIST version 1.1 by both investigators and radiology specialists. An independent central radiological review was not performed. The first documented complete or partial responses should be confirmed after at least 4 weeks. Patients who had first documented disease progression but were considered clinically stable at the investigator's discretion were allowed to continue treatment until progression was confirmed at a subsequent tumor assessment conducted 4 weeks later. Survival follow-up was conducted every three months, either through clinic visits or telephone. All AEs were documented until 90 days after the last administration and graded according to the National Cancer Institute Common Terminology Criteria for Adverse Events (version 5.0).

### Single-cell preparation and library construction

Pre-treatment lung tumor tissues were processed using the Miltenyi Human Tumor Dissociation Kit to obtain single-cell suspensions. Tissues were subjected to mechanical and enzymatic dissociation with the gentleMACS™ Dissociator in combination with the enzymes provided in the Kit. The dissociated tissues were filtered sequentially through 70-μm and 30-μm filters to remove larger debris and impurities. If many erythrocytes were present in the cell suspension, erythrocyte lysate was used to further remove these cells. In addition, dead cells were removed using the Dead Cell Removal Kit to increase the percentage of viable cells. Following the preparation of cell suspension, quality control was performed using the Luna-FL automated cell counter to detect cell viability and concentration. To ensure the quality of the subsequent experiments, the cell viability was required to be maintained above 80% and the concentration was adjusted to 700–1200 cells/μL. In single-cell RNA sequencing, the prepared single-cell suspensions were then loaded into the Chromium Controller via the Chromium Single Cell 3′ Reagent Kits (version 3.1, 10x Genomics, CA, USA) to generate gel beads-in-emulsion (GEMs). After the completion of single-cell reverse transcription in GEMs, the cDNA was purified and amplified by SPRI magnetic beads. During the construction of the library, the quality and concentration of the cDNA were assessed using the Agilent Bioanalyzer and the Qubit dsDNA Assay Kit. Subsequent steps, including cDNA fragmentation, adapter ligation, and sample-indexed PCR, were then performed to construct a high-quality library, which were suitable for sequencing on the Illumina NovaSeq platform.

### Processing and quality control of single-cell sequencing data

The raw sequencing data were processed using the Cellranger toolkit (version 5.0.1, 10X Genomics) according to the standard pipeline, with the human reference genome hg38 (refdata-gex-GRCh38-2020-A). The cell expression matrices generated for each sample were used as input for the R package Seurat (version 5.1.0, R version 4.3.3).^24^ Subsequently, the single-cell transcriptomic profiles from seven samples were subjected to quality control, data integration, and clustering using the Seurat package. Low-quality cells were filtered out by retaining genes expressed in at least three cells, removing cells with fewer than 300 detected genes, and excluding cells with mitochondrial gene content greater than 15%. After filtering, a total of 73,408 cells were retained for downstream analysis. Batch effects were corrected using the Harmony integration algorithm, and highly variable genes were selected according to the default Seurat method. The ScaleData function was applied to regress out the effect of mitochondrial gene percentage, and each gene was scaled to unit variance. The first 15 principal components were used to construct the neighborhood graph, and clustering was performed using the Louvain algorithm with a resolution parameter of 0.5. Dimensionality reduction and visualization were ultimately performed using the uniform manifold approximation and projection (UMAP). The ratio of observed to expected cell numbers (Ro/e) was calculated for each cluster across different tissues to quantify their tissue preference.^25^, ^26^

### Next-generation sequencing

A total of 26 formalin-fixed paraffin-embedded (FFPE) tissue samples were obtained from 26 patients between 2021 and 2024. Paired peripheral blood samples were simultaneously collected to exclude germline mutations. Genomic analyses were performed on tumor tissues containing at least 20% tumor cells and their paired peripheral blood samples. Genomic DNA was extracted using the GeneRead DNA FFPE Kit (Qiagen, Hilden, Germany) and the DNA Blood Mini Kit (Qiagen, Hilden, Germany), respectively. The extracted DNA was amplified, purified, and analyzed with the YuceOne ICIs extensive targeted panel (1267 genes) in pathologists (CAP)-authenticated biotechnique laboratory (Yucebio, Shenzhen, China). Sequencing reads were filtered using SOAPnuke (v1.5.6), and reads with >10% N bases and/or >10% bases with a quality score <20 were removed. Somatic single nucleotide variants (SNVs) and insertions/deletions (InDels) were detected using VarScan (v2.4). Potential false-positive variants were further filtered using the in-house methods. Functional annotation of tumor mutations was performed using SnpEff (v4.3). Copy number variations (CNVs) were identified using CNVkit (v0.8.1) by comparing the exome-wide profiles between tumor and paired blood. Allele-specific copy number and tumor purity were assessed with the ascatNgs (v3.1.0). Tumor mutational burden (TMB) was defined as the number of nonsynonymous mutations and indels per megabase (Mb) of coding region sequenced. Tumor neoantigen burden (TNB) was defined as the number of mutations predicted to generate neoantigens per Mb.

### Outcomes

The primary endpoint was investigator-assessed objective response rate (ORR), defined as the proportion of patients with complete or partial response according to RECIST version 1.1. Secondary endpoints were disease control rate (DCR; defined as the proportion of patients with complete or partial response, or stable disease according to RECIST version 1.1), duration of response (DoR; defined as the time from the first documented response until disease progression or death from any cause, whichever occurred earlier), progression-free survival (PFS; defined as the time from the initiation of study treatment to the first documented disease progression or death from any cause, whichever occurred earlier), OS (defined as the time from the initiation of study treatment to death from any cause), quality of life (QoL), and safety. The genomic characteristics of PSC were exploratory endpoints.

### Statistics analysis

This study adopted Simon's minimax two-stage design, with a one-sided type-I-error of 5% and a statistical power of 80%. According to previous data,^4^, ^27^ the ORR of 35% was assumed for camrelizumab plus famitinib, whereas the response rate less than 15% was regarded as insufficient activity for further investigation in patients with PSC. In the first stage, 15 patients were required to be enrolled and if three or more patients achieved a complete or partial response, the trial would proceed to the second stage with the enrollment of additional 13 evaluable patients. In total, 28 evaluable patients were required, and the combination would be deemed promising if at least eight patients achieved a complete or partial response.

All patients who received at least one dose of study drug were included in the full analysis set (FAS), which was the primary analysis population in this study. Patients in the FAS who had no major protocol deviations or deviations that significantly affected the efficacy outcomes were included in the per-protocol set (PPS). All treated patients who had at least one post-baseline tumor assessment according to RECIST version 1.1 were included in the efficacy analysis set (EAS). Safety was assessed in all as-treated patients (safety set, SS). Descriptive statistics were employed, with continuous variables presented as median and range as well as categorical variables as frequencies and percentages. ORR and DCR were calculated along with their corresponding 95% Clopper-Pearson confidence intervals (CIs). Time-to-event endpoints, including PFS, DoR and OS, were analyzed using Kaplan–Meier method, and the 95% CIs for the median time and survival probability at specific timepoints were calculated based on Brookmeyer-Crowley method and Greenwood method, respectively. Post-hoc subgroup analyses for ORR were based on patient characteristics at baseline. QoL was assessed using the EORTC QLQ-C30 questionnaire in patients who had completed baseline assessment and at least one postbaseline assessment. Higher scores on the global health status and functional scales indicate better QoL, whereas higher scores on the symptom scales indicate a heavier burden. A change of 10 points or more from baseline was considered clinically meaningful. Spearman correlation analysis was used to explore the association between the relative proportions of T-cell subpopulations and the best change in target lesion size from baseline. All statistical analyses were performed using SAS software (version 9.4). The CAPSTONE trial is registered with ClinicalTrials.gov, NCT04888429.

### Role of the funding source

The National Science and Technology Major Project of the Ministry of Science and Technology of China, the National Natural Science Foundation of China, the Innovation Research Group Program of the Natural Science Foundation of Hubei Province, and the Hubei Provincial Health Science and Technology Innovation and Achievement Transformation Project funded the trial, but had no role in the study design, data collection, data analysis, data interpretation, or writing of the clinical study report. Jiangsu Hengrui Pharmaceuticals Co., Ltd. provided the study drugs camrelizumab and famitinib, and participated in study design, data collection, and data analysis, but had no role in data interpretation, or writing of the report.

## Results

### Patients and treatments

Between August 4, 2021 and March 21, 2024, a total of 35 patients were screened, of whom 28 eligible patients were treated with camrelizumab plus famitinib and were included in the FAS and SS (Fig. 1 and Supplementary Table S1). Since no patients had major protocol deviations, the PPS was identical to the FAS. One patient had no post-baseline tumor assessment and was therefore excluded from the EAS, with 27 patients included. In the FAS, the median age was 63 years (range 45–77), and 27 patients (96.4%) were male. The vast majority (96.4%) had an Eastern Cooperative Oncology Group performance status of 1, and 19 (67.9%) had stage IV disease at enrollment. Baseline characteristics are summarized in Table 1.

**Fig. 1:**
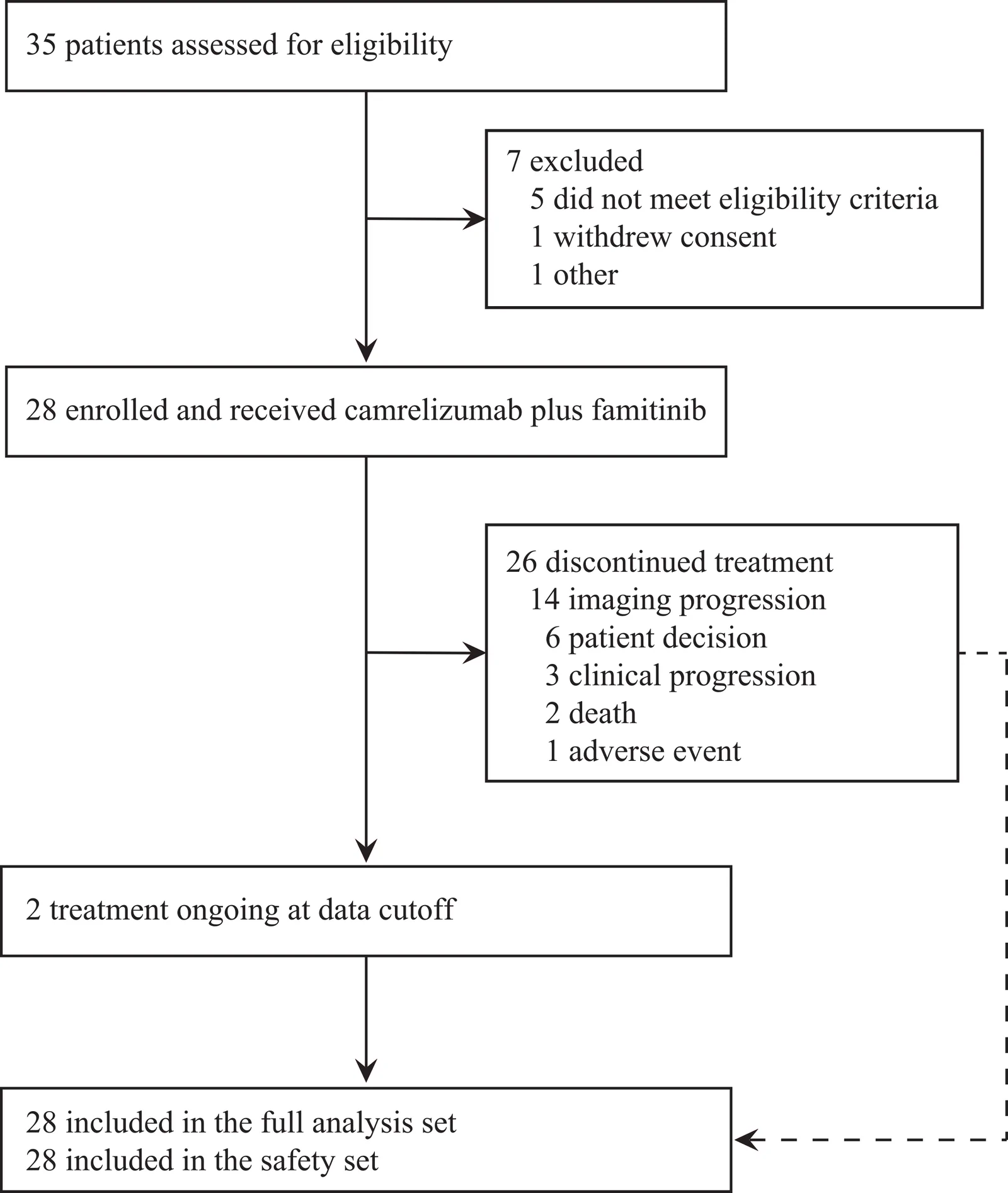
Trial profile.

**Table 1:** Baseline characteristics in the FAS.

	Patients (*n* = 28)
Age (years), median (range)	63 (45–77)
Sex	
Male	27 (96.4)
Female	1 (3.6)
Smoking status	
Never	9 (32.1)
Former smoker	16 (57.1)
Current smoker	3 (10.7)
ECOG PS	
0	1 (3.6)
1	27 (96.4)
Number of metastatic organs	
0	9 (32.1)
1–2	13 (46.4)
≥ 3	6 (21.4)
Clinical stage	
IIIB	7 (25.0)
IIIC	2 (7.1)
IV	19 (67.9)
Histological type	
Spindle cell carcinoma	15 (53.6)
Pleomorphic carcinoma	7 (25.0)
Carcinosarcoma	4 (14.3)
Pulmonary blastoma	1 (3.6)
Giant cell carcinoma	1 (3.6)
PD-L1 expression	
< 1	6 (21.4)
1–50	9 (32.1)
≥ 50	5 (17.9)
Unknown	8 (28.6)
TMB	
Median (IQR)	7.2 (3.4, 10.3)
TMB-low	2 (7.1)
TMB-middle	8 (28.6)
TMB-high	4 (14.3)
Unknown	14 (50.0)

ECOG PS, Eastern Cooperative Oncology Group performance status; FAS, full analysis set; IQR, interquartile range; PD-L1, programmed cell death ligand-1; TMB, tumor mutational burden.

Data are *n* (%), unless otherwise specified.

As of data cutoff (April 7, 2026), the median follow-up was 18.1 months (interquartile range 8.0–28.9). Two (7.1%) of the 28 patients were undergoing study treatment and 26 (92.9%) patients had discontinued treatment, mainly due to imaging progression (14 [50.0%]; Fig. 1). The median number of treatment cycles was 7.5 (range 1–58) for camrelizumab and 6.5 (range 1–61) for famitinib, respectively.

### Efficacy

In the first stage, seven (46.7%) of the 15 patients enrolled had a confirmed partial response, surpassing the predetermined threshold of three responders, thereby allowing the trial to smoothly advance to the second stage. Of all 28 patients enrolled, 12 (42.9%) had a confirmed partial response, with the confirmed ORR of 42.9% (95% CI 24.5–62.8; Table 2). Post-hoc subgroup analyses for ORR are shown in Supplementary Fig. S1. The median DoR among these 12 patients was 16.0 months (95% CI 5.0–29.9; Supplementary Fig. S2A). Additional 12 (42.9%) patients had stable disease, with the DCR of 85.7% (95% CI 67.3–96.0). In the EAS, the confirmed ORR was 44.4% (95% CI 25.5–64.7) and the DCR was 88.9% (95% CI 70.8–97.7; Supplementary Table S2). Of the 27 patients who had evaluable tumor assessments, 24 (88.9%) had a decrease from baseline in sum of target lesion size (Supplementary Fig. S2B). The duration of treatment for each patient is illustrated in Supplementary Fig. S2C.

**Table 2:** Confirmed tumor response in the FAS.

	Patients (*n* = 28)
Confirmed best overall response	
Complete response	0
Partial response	12 (42.9)
Stable disease	12 (42.9)
Progressive disease	3 (10.7)
Not evaluable	1 (3.6)
Objective response rate	12 (42.9%, 24.5–62.8)
Disease control rate	24 (85.7%, 67.3–96.0)

CI, confidence interval; FAS, full analysis set.

Data are *n* (%) or *n* (%, 95% CI).

At the data cutoff, disease progression or death occurred in 26 (92.9%) of the 28 patients. The median PFS (mPFS) was 7.1 months (95% CI 3.5–12.5; Fig. 2A). The 6-, 12- and 18-month PFS rates were 57.1% (95% CI 37.1–72.9), 35.7% (95% CI 18.9–53.0), and 28.6% (95% CI 13.5–45.6), respectively. Post-hoc subgroup analyses showed that the mPFS was 7.8 months (95% CI 1.4–26.3) in patients with spindle cell carcinoma, compared with 5.2 months (95% CI 2.6–12.6) in those with other histological types (Supplementary Fig. S3). Nineteen (67.9%) patients had died, with the median OS of 18.1 months (95% CI 10.3–28.1; Fig. 2B). The 6-month OS rate was 85.7% (95% CI 66.3–94.4), as well as the 12- and 18-month rates were 60.7% (95% CI 40.4–76.0) and 53.6% (95% CI 33.8–69.8), respectively. Post-hoc subgroup analyses of PFS and OS according to PD-L1 expression level are shown in Supplementary Fig. S4.

**Fig. 2:**
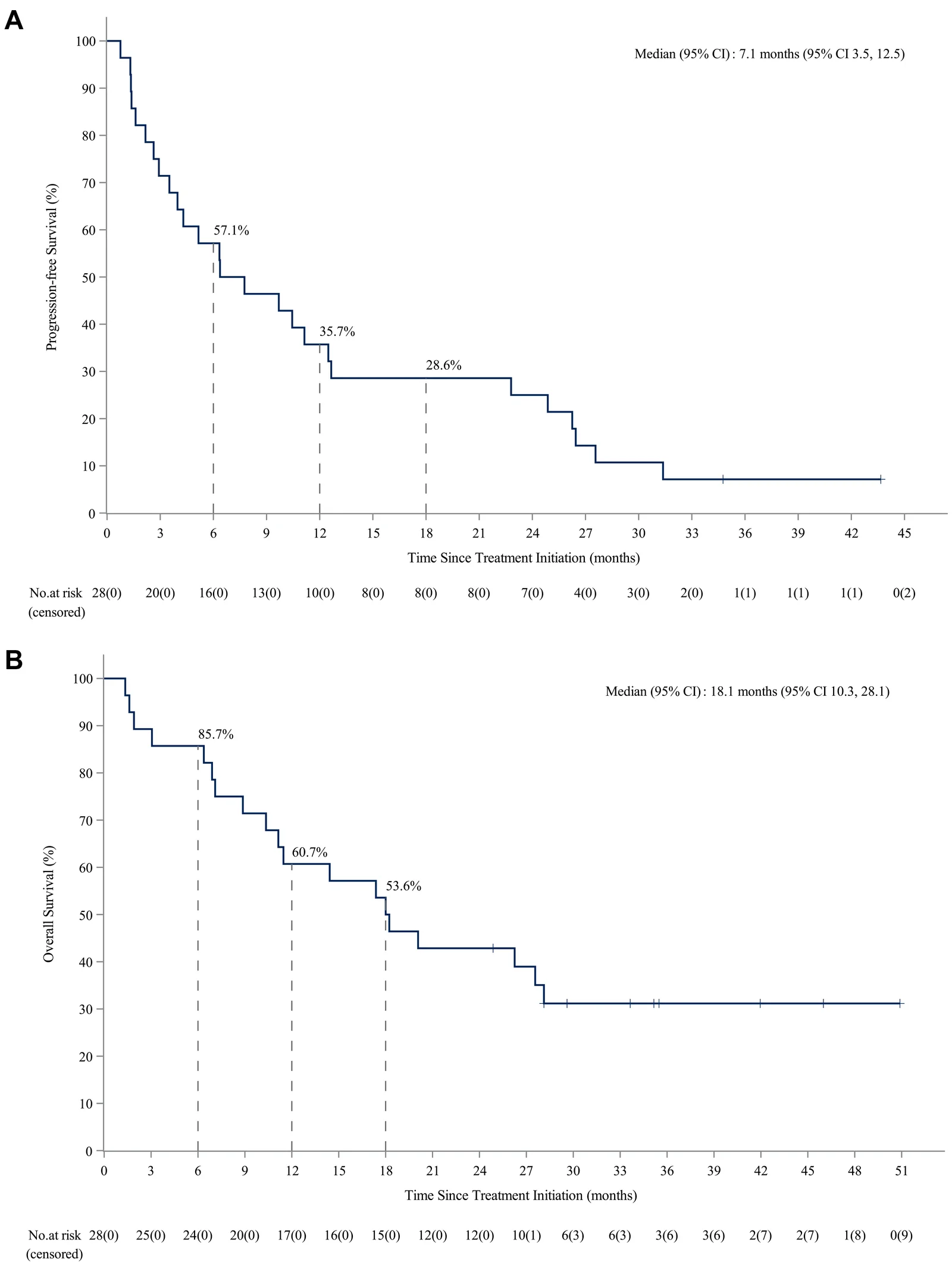
Kaplan–Meier estimates for progression-free survival (A), and overall survival (B) in the FAS. CI, confidence interval; FAS, full analysis set.

In the EAS, the mPFS was 7.8 months (95% CI 3.5–12.6), with the 6-, 12- and 18-month PFS rates of 59.3% (95% CI 38.6–75.0), 37.0% (95% CI 19.6–54.6), and 29.6% (95% CI 14.1–47.0), respectively (Supplementary Fig. S5A). The median OS was 18.2 months (95% CI 10.3-not estimable), with the 6-, 12- and 18-month OS rates of 88.9% (95% CI 69.4–96.3), 63.0% (95% CI 42.1–78.1), and 55.6% (95% CI 35.2–71.8), respectively (Supplementary Fig. S5B).

### Subsequent anticancer therapies

Among the 20 patients who experienced disease progression, 10 (50.0%) received subsequent anticancer treatment, six (30.0%) did not receive subsequent treatment, and four (20.0%) had unknown subsequent treatment status. The detailed subsequent anticancer regimens are presented in Supplementary Table S3.

### Quality of life

Overall, global health status showed a clinically meaningful improvement from baseline to cycle 17, with the mean score increasing from 61.9 to 78.6 (Supplementary Fig. S6 and Supplementary Table S4). All functional scale scores remained generally stable during treatment. Clinically meaningful improvements from baseline to cycle 17 were also observed in symptom scores for pain (mean score, 21.2 at baseline vs. 9.5 at cycle 17), insomnia (25.6 vs. 9.5), and constipation (19.2 vs. 4.8). The other symptom scale scores remained stable, except for diarrhea, for which the score deteriorated from 5.1 at baseline to 28.6 at cycle 17 (Supplementary Table S4).

### Safety

Treatment-related AEs (TRAEs) of any grade were reported in 27 (96.4%) of the 28 patients, with the most common events being proteinuria (17 [60.7%]), hypertension (14 [50.0%]), decreased neutrophil count (14 [50.0%]), decreased platelet count (13 [46.4%]), and increased aspartate aminotransferase (11 [39.3%]; Table 3). Grade 3 or more TRAEs were observed in 10 (35.7%) patients, mainly composed of decreased platelet count (four [14.3%]) and decreased neutrophil count (three [10.7%]).

**Table 3:** Treatment-related adverse events.

Treatment-related adverse events	Patients (*n* = 28)	
	Any grade	Grade ≥ 3
Any events	27 (96.4)	10 (35.7)
Proteinuria	17 (60.7)	2 (7.1)
Hypertension	14 (50.0)	2 (7.1)
Neutrophil count decreased	14 (50.0)	3 (10.7)
Platelet count decreased	13 (46.4)	4 (14.3)
Aspartate aminotransferase increased	11 (39.3)	2 (7.1)
White blood cell count decreased	9 (32.1)	0
Hand-foot syndrome	9 (32.1)	0
Hematuria	9 (32.1)	0
Alanine aminotransferase increased	8 (28.6)	1 (3.6)
Anemia	8 (28.6)	0
Hypothyroidism	5 (17.9)	0
Diarrhea	4 (14.3)	0
Hyperthyroidism	4 (14.3)	0
Decreased appetite	4 (14.3)	0
Haematochezia	3 (10.7)	0
Fever	3 (10.7)	0
Reactive cutaneous capillary endothelial proliferation	3 (10.7)	0
Hemoptysis	3 (10.7)	1 (3.6)
Dental ulcer	3 (10.7)	0
Weakness	3 (10.7)	0
Blood creatinine increased	3 (10.7)	0
Unknown death	2 (7.1)	2 (7.1)

Data are *n* (%). The table shows treatment-related adverse events of any grade occurring in at least 10% of patients, and all grade 3 or more events occurring.

TRAEs led to dose interruptions of famitinib in 16 (57.1%) of the 28 patients, and the most frequent reasons for interruptions were proteinuria (seven [25.0%]), decreased platelet count (six [21.4%]), and increased aspartate aminotransferase (five [17.9%]). TRAEs led to dose reductions of famitinib in four (14.3%) patients, mainly due to decreased white blood cell count (two [7.1%]; Supplementary Table S5). Three (10.7%) patients discontinued camrelizumab: one due to increased aspartate aminotransferase and alanine aminotransferase, and the other two owing to decreased platelet count and increased alanine aminotransferase, respectively; three (10.7%) discontinued famitinib: one because of proteinuria and two due to hemoptysis.

Immune-related adverse events of any grade were observed in 11 (39.3%) patients, with the most common being increased aspartate aminotransferase (five [17.9%]), increased alanine aminotransferase (four [14.3%]), and hypothyroidism (four [14.3%], Supplementary Table S6). Only one (3.6%) patient experienced a grade 3 immune-related adverse event, namely increased aspartate aminotransferase.

Serious TRAEs occurred in six (21.4%) patients (Supplementary Table S7). Two patients died due to unknown cause, which was assessed to be possibly related to study treatment by the investigator. The two male patients were aged 70 and 65 years, respectively; both had stage IV disease, with baseline target lesion diameters of 135 mm and 236 mm, respectively. They developed grade 4 decreased platelet count after one and two cycles of study treatment, respectively, and study treatment was discontinued. In one patient, this grade 4 event did not resolve after symptomatic treatment. The patient remained in poor condition and was unable to eat during hospitalization, and died 40 days after initiation of study treatment. The other patient was transferred to another hospital shortly after admission at the family's request, and subsequent clinical information was unavailable. Telephone follow-up confirmed that this patient died 29 days after the second cycle of treatment. The cause of death was unknown in both cases as no autopsy was performed. Given the metastatic disease and substantial baseline tumor burden, death due to disease progression could not be excluded. However, because of the temporal relationship among treatment exposure, grade 4 AE, and death, a treatment-related contribution could also not be ruled out. Accordingly, both deaths were assessed by the investigators as possibly related to study treatment.

### Genomic alteration and single-cell sequencing data

Among 26 patients who had available genomic alteration data, the most frequent mutations were TP53 (69%), EGFR (19%), KRAS (19%), and PTEN (19%; Supplementary Fig. S7A). The specific variants of five patients with EGFR alterations are summarized in Supplementary Table S8, and none were EGFR-sensitizing mutations specified in the exclusion criteria. Neither tissue TMB nor TNB demonstrated a significant association with PFS or OS (Supplementary Fig. S7B).

As an exploratory translational analysis, single-cell RNA sequencing was performed on pretreatment lung tumor tissues obtained from seven patients (Supplementary Fig. S8A and Supplementary Table S9). A total of 73,408 cells were analyzed and classified into 28 clusters (Fig. 3A), which were annotated as seven major cell types: epithelial cells, fibroblasts, endothelial cells, T/NK cells, myeloid cells, B cells, and mast cells (Fig. 3B and C). Patients were stratified into <mPFS and ≥mPFS groups according to the mPFS (Supplementary Table S9). Descriptive comparisons between groups showed broadly similar distributions of the major cell types (Supplementary Fig. S8B–8E).

**Fig. 3:**
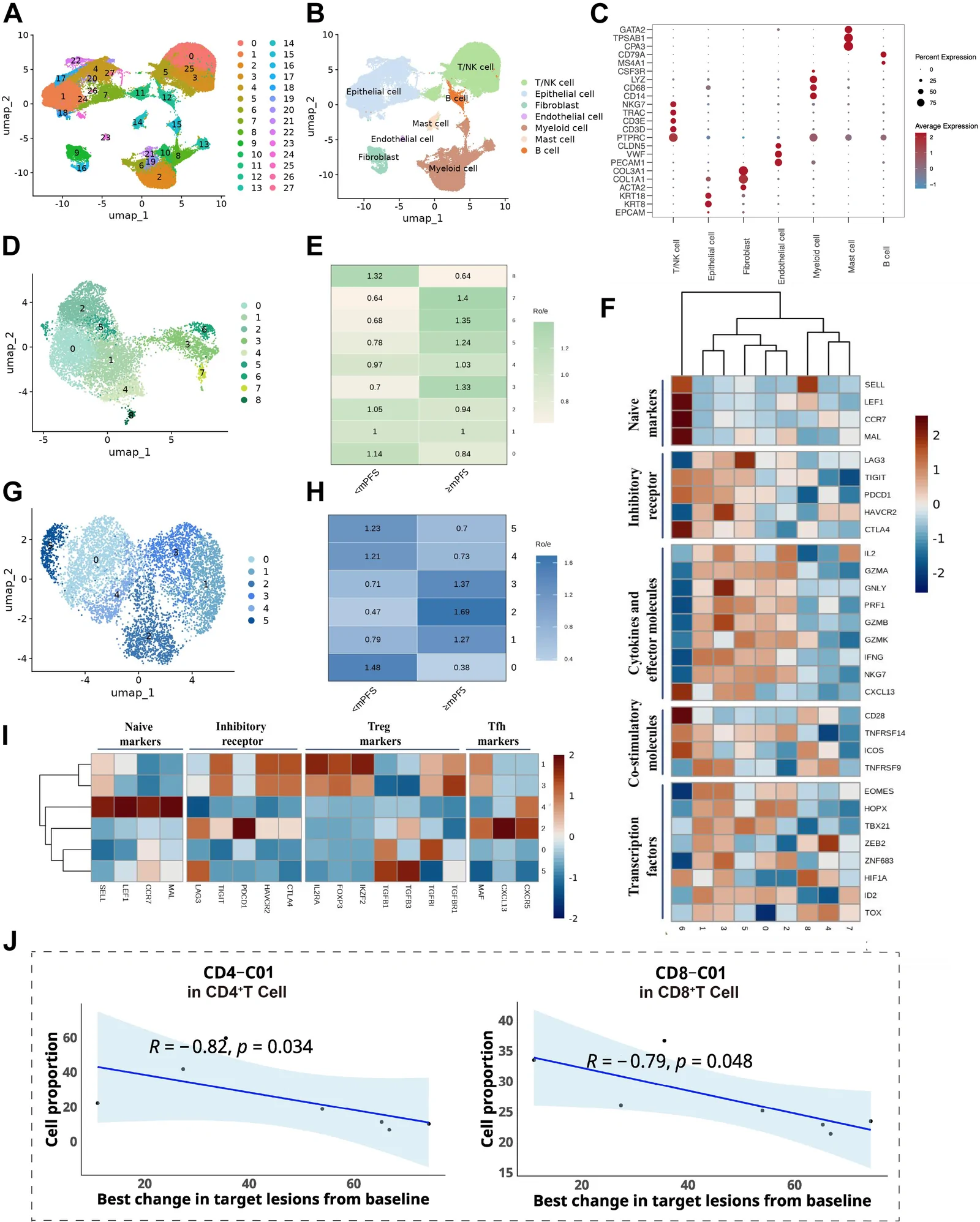
T-Cell infiltration and functional differences in patients with different treatment responses. (A) UMAP plot showing the 28 main cell types identified from cancer tissues (*n* = 7), with a total of 73,408 cells. (B) UMAP plot of cancer tissue cells, colored by cell type. (C) Dot plot displaying the expression levels of marker genes across 7 major cell types. (D) UMAP plot highlighting the clusters of CD8+ T cells, color-coded by cell type. (E) Heatmap depicting tissue distribution preferences of CD8+ T cells in patients with different treatment responses (R_O/E_). (F) Heatmap illustrating functional phenotype variations of CD8+ T cells. (G) UMAP plot highlighting the clusters of CD4+ T cells, color-coded by cell type. (H) Heatmap displaying tissue distribution preferences of CD4+ T cells across patients with different treatment responses (R_O/E_). (I) Heatmap categorizing different CD4+ T cell subsets. (J) Correlation analysis assessing the relationship between the best change in target lesions from baseline and the proportion of the CD4-C0 subgroup and CD8-C0 subgroup using Spearman correlation. mPFS, median progression-free survival.

Subclustering of T/NK cells identified CD4^+^ T cells, CD8^+^ T cells, and NK cells (Supplementary Fig. S9A). CD8^+^ T cells from the ≥mPFS group showed relatively higher expression of PDCD1, IFNG, and MKI67, together with higher functional signature scores (Supplementary Fig. S9B–9D). Further subclustering revealed differences in the relative proportions of several CD4^+^ and CD8^+^ T-cell subpopulations between the two groups (Fig. 3D–I).

We next explored the association between T-cell subpopulations and changes in target lesion size (Supplementary Fig. S10). Exploratory patient-level analyses showed inverse associations between the proportions of the cluster 0 CD4^+^ and CD8^+^ T cell subpopulations (Fig. 3J). Cluster 0 CD8^+^ T cells preferentially expressed CXCR4 and DUSP2, whereas cluster 0 CD4^+^ T cells expressed ANXA1 and IL7R (Supplementary Fig. S11A). MultiNicheNet analysis showed differences in predicted intercellular communication patterns between the two groups, with relatively greater predicted contributions from myeloid cells in the ≥mPFS group and cluster 0 CD4+ T cells in the <mPFS group (Supplementary Fig. S11B).

## Discussion

To our knowledge, this CAPSTONE trial is one of the few prospective trials specifically evaluating combination therapy with a PD-1 inhibitor and an antiangiogenic agent in the first-line setting for patients with locally advanced or metastatic PSC. This trial met the primary endpoint, demonstrating that camrelizumab plus famitinib resulted in a confirmed ORR of 42.9% for all enrolled patients, markedly exceeding the target value of 35% established in the study design. The majority of TRAEs were managed by means of dose adjustments or symptomatic treatment.

Evidence for first-line ICIs-based therapies in PSC has gradually emerged. Several retrospective studies have shown that first-line immunotherapy, mainly ICI monotherapy or ICIs combined with chemotherapy, achieved an ORR of 37.5%–70.6%, a mPFS of 3.45–10.3 months, and a median OS of 13.11–24.9 months in patients with advanced PSC.^15^, ^28^, ^29^, ^30^, ^31^, ^32^, ^33^ Nevertheless, available retrospective data have not demonstrated that adding chemotherapy to ICI-based treatment further improves clinical outcomes, suggesting that the additional benefit of chemotherapy in this setting may be limited.^28^, ^30^, ^34^ In addition, a retrospective study conducted by Xie et al. reported that PSC patients treated with ICIs plus anti-angiogenic therapy had a higher ORR (45.5% vs. 33.8%) and longer PFS (13.1 vs. 7.6 months, HR 0.53, 95% CI 0.30–0.92, P = 0.044) compared to those without anti-angiogenic therapy.^15^ Moreover, ICIs plus anti-angiogenic therapy yielded superior ORR (47.6% vs. 0%) and PFS (24.4 vs. 8.8 months) compared to the combination of ICIs with anti-angiogenic therapy and chemotherapy in this patient population.^15^ Recently, a single-center phase 2 study reported that first-line tislelizumab plus anlotinib yielded an ORR of 55.17%, a median DoR of 9.80 months, an mPFS of 9.40 months, and a median OS of 14.37 months in advanced PSC.^35^ Consistent with these findings, our regimen with camrelizumab plus famitinib also exhibited antitumor activity, with the confirmed ORR of 42.9%, median DoR of 16.0 months, mPFS of 7.1 months, and median OS of 18.1 months. Collectively, these prospective findings further suggest the feasibility of first-line chemotherapy-free strategy with ICIs plus antiangiogenic agents for advanced PSC. However, the small sample sizes and single-arm designs of these studies limit their capacity to establish the added value of antiangiogenic agents in the combination regimen. Therefore, further validation in larger randomized controlled studies is warranted.

The genomic alteration profile observed in our study, including frequent alterations in TP53, EGFR, KRAS, and PTEN, was broadly consistent with previous reports.^11^, ^36^, ^37^ Given the molecular and immunological heterogeneity of PSC, we further explored tumor immune features potentially associated with treatment outcomes. In the exploratory scRNA-seq analysis, specific CD4^+^ and CD8^+^ T-cell subpopulations showed different distribution patterns between the <mPFS and ≥mPFS groups, and the proportions of cluster 0 CD4^+^ and CD8^+^ T cells showed a potential inverse association with the best change from baseline in target lesion size. Cluster 0 CD8^+^ T cells were characterized by relatively higher expression of CXCR4 and DUSP2, whereas cluster 0 CD4^+^ T cells showed relatively higher expression of ANXA1 and IL7R. Previous studies have implicated these genes in T-cell trafficking, exhaustion and immunosuppressive signaling.^38^, ^39^, ^40^, ^41^, ^42^, ^43^ MultiNicheNet analysis further indicated potential differences in predicted intercellular communication patterns between the two groups. Taken together, these exploratory findings suggest that distinct CD8^+^ and CD4^+^ T-cell states might be associated with treatment response in PSC, and their potential roles in immunosuppression and therapeutic resistance warrant further investigation.

The safety profile of camrelizumab plus famitinib was generally consistent with that previously reported for each agent and their combination.^44^, ^45^, ^46^ The most common non-hematological TRAEs, hypertension and proteinuria, were mostly associated with famitinib and were predominantly grade 1–2, which was comparable to previously reported incidence.^22^, ^23^, ^44^ Four patients reduced famitinib dose from the perspective of mitigating toxicities and maximizing patient benefit. Two patient deaths (of unknown cause) were assessed by the investigators as possibly to study treatment. Both patients had a heavy tumor burden, with the target lesion diameters of respective 135 mm and 236 mm, and died within 40 and 29 days following one and two cycles of treatment, respectively. But we were unable to exclude a correlation between treatment and mortality outcome due to the lack of evidence of disease progression. No novel or unexpected TRAEs were identified.

This study has several limitations. First, a single-arm study design was employed in view of its exploratory nature; however, the lack of a control group and independent central review may have introduced selection, reporting and assessment biases. Second, the small sample size resulted in wide CIs around efficacy estimates and precluded the drawing of definitive conclusions. Therefore, the findings should be interpreted with caution and require confirmation in larger studies. Third, patient recruitment was imbalanced across participating centers, with more than half of the patients enrolled at the principal institution but none enrolled at two participating centers. Consequently, the study population may have been more reflective of the patient characteristics at the principal institution, potentially limiting the representativeness of the study population. Fourth, the small subgroup sizes according to PD-L1 status, together with 28.6% of patients with unknown PD-L1 status due to insufficient tumor tissue for testing, limited the robustness of the subgroup analysis. Therefore, these findings are insufficient to establish PD-L1 expression as a predictive biomarker and may affect the interpretation of efficacy. In addition, the single-cell sequencing was performed only in seven patients, which was exploratory in nature. The identified prognostic-related biomarkers should therefore be considered hypothesis-generating and warrant validation in larger cohorts. However, considering that PSC is a rare disease with a low prevalence and that historical data available on treatment in the first-line setting are scarce, our findings offer several additional valuable insights. Future larger, prospective, randomized controlled trials are warranted to further evaluate this combination for this rare population and determine the contribution of famitinib. Further validation of potential biomarkers may also help refine patient selection.

More recently, several promising new drugs have emerged, such as bispecific antibodies targeting both PD-(L)1 and VEGF. Ivonescimab, a PD-1 and VEGF bispecific antibody, has shown promising efficacy in patients with advanced NSCLC.^47^, ^48^ Another PD-L1/VEGF-A bispecific pumitamig (BNT327) is being investigated for NSCLC (NCT06841055, NCT07361510). Besides, the high expression of TROP2 in PSC provides a potential rationale for investigating TROP2-directed antibody-drug conjugates.^49^ Future studies evaluating these emerging agents in PSC may provide additional therapeutic options for this rare disease.

In conclusion, the CAPSTONE trial indicated antitumor activity of camrelizumab plus famitinib as first-line therapy in patients with locally advanced or metastatic PSC. Our findings suggest that this chemotherapy-free strategy warrants further investigation as a potential first-line regimen for this patient population.

## Contributors

QC contributed to the design of the study. QC, JJ, BY, XL, SC, QS, HW, JW, and FY recruited patients and collected the data. YW, FS and HK contributed to data analysis. JJ and YW participated in data interpretation. ZJ and BZ contributed to biomarker analysis. QC provided the funding support, coordinated and supervised the study. JJ and YW drafted the manuscript. All authors reviewed the manuscript and approved the final version. QC had final responsibility for the decision to submit for publication. All authors accessed and verified the data.

## Data sharing statement

Deidentified individual patient data are available from the corresponding author on reasonable request.

## Declaration of interests

FS and HK report being employed by Jiangsu Hengrui Pharmaceuticals Co., Ltd. All other authors declare no competing interests.

## Appendix A Supplementary data

Supplementary Data

Study Protocol

## References

[1] Travis W.D., Brambilla E., Nicholson A.G. (2015). The 2015 World Health Organization classification of lung tumors: impact of genetic, clinical and radiologic advances since the 2004 classification. J Thorac Oncol.

[2] Rahouma M., Kamel M., Narula N. (2018). Pulmonary sarcomatoid carcinoma: an analysis of a rare cancer from the Surveillance, Epidemiology, and End Results database. Eur J Cardiothorac Surg.

[3] Steuer C.E., Behera M., Liu Y. (2017). Pulmonary sarcomatoid carcinoma: an analysis of the national cancer data base. Clin Lung Cancer.

[4] Vieira T., Girard N., Ung M. (2013). Efficacy of first-line chemotherapy in patients with advanced lung sarcomatoid carcinoma. J Thorac Oncol.

[5] Ung M., Rouquette I., Filleron T. (2016). Characteristics and clinical outcomes of sarcomatoid carcinoma of the lung. Clin Lung Cancer.

[6] Oizumi S., Takamura K., Harada T. (2022). Phase II study of carboplatin-paclitaxel alone or with bevacizumab in advanced sarcomatoid carcinoma of the lung: HOT1201/NEJ024. Int J Clin Oncol.

[7] Novello S., Kowalski D.M., Luft A. (2023). Pembrolizumab plus chemotherapy in squamous non-small-cell lung cancer: 5-Year update of the phase III KEYNOTE-407 study. J Clin Oncol.

[8] Zhou C., Chen G., Huang Y. (2024). 61P first-line camrelizumab (Camre) plus carboplatin (Carbo) and pemetrexed (Pem) for advanced non-squamous NSCLC: 5-yr outcomes of CameL phase III study. ESMO Open.

[9] Vieira T., Antoine M., Hamard C. (2016). Sarcomatoid lung carcinomas show high levels of programmed death ligand-1 (PD-L1) and strong immune-cell infiltration by TCD3 cells and macrophages. Lung Cancer.

[10] Velcheti V., Rimm D.L., Schalper K.A. (2013). Sarcomatoid lung carcinomas show high levels of programmed death ligand-1 (PD-L1). J Thorac Oncol.

[11] Yang Z., Xu J., Li L. (2020). Integrated molecular characterization reveals potential therapeutic strategies for pulmonary sarcomatoid carcinoma. Nat Commun.

[12] Chang Y.L., Yang C.Y., Lin M.W., Wu C.T., Yang P.C. (2016). High co-expression of PD-L1 and HIF-1 alpha correlates with tumour necrosis in pulmonary pleomorphic carcinoma. Eur J Cancer.

[13] Acuna-Pilarte K., Koh M.Y. (2025). The HIF axes in cancer: angiogenesis, metabolism, and immune-modulation. Trends Biochem Sci.

[14] Tsao A.S., Wei W., Kuhn E. (2011). Immunohistochemical overexpression of platelet-derived growth factor receptor-beta (PDGFR-beta) is associated with PDGFRB gene copy number gain in Sarcomatoid non-small-cell lung cancer. Clin Lung Cancer.

[15] Xie M., Chu T., Dong X. (2024). Heterogeneity in advanced pulmonary sarcomatoid carcinoma and its efficacy to immune checkpoint inhibitors. Eur J Cancer.

[16] Wen Y., Dong Y., Yi L. (2023). Anlotinib combined with pembrolizumab as first-line treatment for advanced pulmonary sarcomatoid carcinoma: a case report and literature review. Front Oncol.

[17] Cai R., Liu Y., Sha H. (2023). A case report: a new promising treatment for pulmonary sarcomatoid carcinoma - Tislelizumab and Anlotinib combined with local radiotherapy. Heliyon.

[18] Zhou C., Chen G., Huang Y. (2021). Camrelizumab plus carboplatin and pemetrexed versus chemotherapy alone in chemotherapy-naive patients with advanced non-squamous non-small-cell lung cancer (CameL): a randomised, open-label, multicentre, phase 3 trial. Lancet Respir Med.

[19] Ren S., Chen J., Xu X. (2022). Camrelizumab plus carboplatin and paclitaxel as first-line treatment for advanced squamous NSCLC (CameL-Sq): a phase 3 trial. J Thorac Oncol.

[20] Xie C., Zhou J., Guo Z. (2013). Metabolism and bioactivation of famitinib, a novel inhibitor of receptor tyrosine kinase, in cancer patients. Br J Pharmacol.

[21] Ren S., Wang X., Han B.H. (2024). First-line treatment with camrelizumab plus famitinib in advanced or metastatic NSCLC patients with PD-L1 TPS >/=1%: results from a multicenter, open-label, phase 2 trial. J Immunother Cancer.

[22] Qu Y.Y., Zhang H.L., Guo H. (2021). Camrelizumab plus famitinib in patients with advanced or metastatic renal cell carcinoma: data from an open-label, multicenter phase II basket study. Clin Cancer Res.

[23] Xia L., Zhou Q., Gao Y. (2022). A multicenter phase 2 trial of camrelizumab plus famitinib for women with recurrent or metastatic cervical squamous cell carcinoma. Nat Commun.

[24] Butler A., Hoffman P., Smibert P., Papalexi E., Satija R. (2018). Integrating single-cell transcriptomic data across different conditions, technologies, and species. Nat Biotechnol.

[25] Zhang L., Yu X., Zheng L. (2018). Lineage tracking reveals dynamic relationships of T cells in colorectal cancer. Nature.

[26] Guo X., Zhang Y., Zheng L. (2018). Global characterization of T cells in non-small-cell lung cancer by single-cell sequencing. Nat Med.

[27] Sun L., Dai J., Chen Y. (2020). Pulmonary sarcomatoid carcinoma: experience from SEER database and Shanghai Pulmonary Hospital. Ann Thorac Surg.

[28] Amrane K., Cabarrou B., Justeau G. (2025). Efficacy of first-line treatments for advanced pulmonary sarcomatoid carcinomas: a real-world analysis. ESMO Open.

[29] Birsen G., Gounant V., Girard N. (2024). Efficacy of first-line immune checkpoint inhibitors in pulmonary sarcomatoid carcinoma. Transl Lung Cancer Res.

[30] Hazama D., Nakahama K., Kodama H. (2024). Effectiveness and safety of immune checkpoint inhibitors alone or in combination with chemotherapy in pulmonary sarcomatoid carcinoma. JTO Clin Res Rep.

[31] Qian X., Wang Y., Liu F. (2022). The efficacy and safety analysis of first-line immune checkpoint inhibitors in pulmonary sarcomatoid carcinoma. Front Immunol.

[32] Wu M., Zhou N., Guan M., Wang Y., Wang Y. (2025). Efficacy of immune checkpoint inhibitors in pulmonary sarcomatoid carcinoma and predictive potential of mutated TP53. Lung Cancer.

[33] Zhou F., Guo H., Zhou X. (2022). Immune checkpoint inhibitors plus chemotherapy in patients with locally advanced or metastatic pulmonary sarcomatoid carcinoma: a multicentric real-world study. Ther Adv Med Oncol.

[34] Hong L., Di Federico A., Liu B. (2025). Distinct clinicogenomic features and immunotherapy associations in pulmonary sarcomatoid carcinoma: a multicenter retrospective study. J Thorac Oncol.

[35] Zeng Z., Huang W., Zeng F. (2026). The efficacy and safety of tislelizumab plus anlotinib as first-line treatment in advanced pulmonary sarcomatoid carcinoma: a single-arm phase II trial. Clin Cancer Res.

[36] Fallet V., Saffroy R., Girard N. (2015). High-throughput somatic mutation profiling in pulmonary sarcomatoid carcinomas using the LungCarta Panel: exploring therapeutic targets. Ann Oncol.

[37] Ullah A., Ahmed A., Yasinzai A.Q.K. (2023). Demographics and clinicopathologic profile of pulmonary sarcomatoid carcinoma with survival analysis and genomic landscape. Cancers (Basel).

[38] Steele M.M., Jaiswal A., Delclaux I. (2023). T cell egress via lymphatic vessels is tuned by antigen encounter and limits tumor control. Nat Immunol.

[39] Seo Y.D., Jiang X., Sullivan K.M. (2019). Mobilization of CD8(+) T cells via CXCR4 blockade facilitates PD-1 checkpoint therapy in human pancreatic cancer. Clin Cancer Res.

[40] Dan L., Liu L., Sun Y. (2020). The phosphatase PAC1 acts as a T cell suppressor and attenuates host antitumor immunity. Nat Immunol.

[41] Bai F., Zhang P., Fu Y. (2020). Targeting ANXA1 abrogates Treg-mediated immune suppression in triple-negative breast cancer. J Immunother Cancer.

[42] Araujo T.G., Mota S.T.S., Ferreira H.S.V., Ribeiro M.A., Goulart L.R., Vecchi L. (2021). Annexin A1 as a regulator of immune response in cancer. Cells.

[43] Kawabe T., Yi J., Sprent J. (2021). Homeostasis of naive and memory T lymphocytes. Cold Spring Harb Perspect Biol.

[44] Qu Y.Y., Sun Z., Han W. (2022). Camrelizumab plus famitinib for advanced or metastatic urothelial carcinoma after platinum-based therapy: data from a multicohort phase 2 study. J Immunother Cancer.

[45] Xu R.H., Shen L., Wang K.M. (2017). Famitinib versus placebo in the treatment of refractory metastatic colorectal cancer: a multicenter, randomized, double-blinded, placebo-controlled, phase II clinical trial. Chin J Cancer.

[46] Ma Y., Cao J., Zhang Y. (2023). Phase I study of camrelizumab in patients with advanced solid tumors. Signal Transduct Target Ther.

[47] Wang L., Luo Y., Ren S. (2024). A phase 1b study of ivonescimab, a programmed cell death protein-1 and vascular endothelial growth factor bispecific antibody, as first- or second-line therapy for advanced or metastatic immunotherapy-naive NSCLC. J Thorac Oncol.

[48] Xiong A., Wang L., Chen J. (2025). Ivonescimab versus pembrolizumab for PD-L1-positive non-small cell lung cancer (HARMONi-2): a randomised, double-blind, phase 3 study in China. Lancet.

[49] Qian X., Luo C., Fang C. (2025). High Trop-2 expression in pulmonary sarcomatoid carcinoma reveals antibody-drug conjugate targeting Trop-2 is a promising therapeutic approach. J Transl Med.

