# Supplementary material for: Camrelizumab plus famitinib as first-line treatment for locally advanced or metastatic pulmonary sarcomatoid carcinomas (CAPSTONE): a multi-center, single-arm, phase 2 trial
Source: eClinicalMedicine. 2026 Oct 24;101:104285. doi: 10.1016/j.eclinm.2026.104285 (PMC13720253; doi:10.1016/j.eclinm.2026.104285)
Supplement: Supplementary file 1 [file mmc2.pdf]

Camrelizumab Plus Famitinib as First-line Treatment in Patients with Advanced or Metastatic Pulmonary Sarcomatoid Carcinoma: A Multi-Center, Single-Arm Study (CAPSTONE)

Protocol Number: MA-PSC-II-001

Protocol Version: 2.0

Version Date: January 17, 2022

**Leading Study Site**	Tongji Hospital Affiliated to Tongji Medical College of Huazhong University of Science and Technology
**Principal Investigator**	Chu Qian
**Sponsor**	Tongji Hospital Affiliated to Tongji Medical College of Huazhong University of Science and Technology

VERSION HISTORY/AMENDMENT HISTORY

Document	Version Date	Summary of Revision
V1.0	December 7, 2020	· Not applicable
V1.1	March 4, 2021	· Removed "Open" from the title<br>· Supplemented rational of dosing regimen design for famitinib malate capsules<br>· Supplemented risk/benefit assessment
V2.0	January 17, 2022	· Updated version number, version date, leading study site, principal investigator and sponsor updates

Table of Contents

Table of Contents **3**

Study Flow Chart **6**

1. Study Background **10**

1.1 Epidemiological Characteristics and Current Treatment Status of Pulmonary Sarcomatoid Carcinoma ***10***

1.2 Pulmonary Sarcomatoid Carcinoma and Immunotherapy ***10***

1.3 Pulmonary Sarcomatoid Carcinoma and Anti-Angiogenic Therapy ***11***

1.4 Synergistic Effect of Anti-Angiogenic Therapy and Immunotherapy ***11***

2. Study Drugs **12**

2.1 Camrelizumab ***12***

2.2 Famitinib Malate Capsules ***12***

2.3 Rationale for the Famitinib Malate Capsules Dosage Regimen Design in the Combination Therapy ***13***

2.4 Risk/Benefit Assessment ***14***

2.4.1 Main Safety Risks 14

2.4.2 Potential Benefits 17

3. Study Objectives and Endpoints **17**

3.1 Study Objectives ***17***

3.2 Study Endpoints ***18***

3.2.1 Primary Endpoint 18

3.2.2 Secondary Endpoints 18

3.2.3 Exploratory Endpoint： 18

4. Study Design **18**

4.1 Study Design Diagram ***18***

4.2 Study Population ***18***

4.2.1 Sample Size Determination 19

4.2.2 Patient Selection 19

4.2.3 Subject Withdrawal Criteria 21

4.2.4 Study Termination Criteria 21

4.3 Treatment Regimen and Dosage ***22***

4.3.1 Treatment Regimen 22

4.3.2 Dosage Regimen 23

4.4 Dose Adjustment and Discontinuation *23*
4.4.1 General Principles of Dose Adjustment 23
4.4.2 Camrelizumab Dose Adjustment 24
4.4.3 Famitinib Dose Adjustment 26
4.5 Concomitant Treatment *28*
4.5.1 Hematologic Support 28
4.5.2 Treatment of Non-Hematologic Toxicities 28
4.5.3 Prohibited Medications During the Study 29
4.6 Study Procedure *30*
4.6.1 Screening Period 31
4.6.2 Treatment Period 32
4.6.3 End of Treatment 33
4.6.4 Safety Follow-Up 34
4.6.5 Survival Follow-up 35
4.7 Drug Management *35*
4.7.1 Drug Management 35
4.7.2 Drug Retrieval and Destruction 35
4.7.3 Study Drug Records 36
5. Clinical Evaluation **36**
5.1 Primary Efficacy Endpoint *36*
5.2 Secondary Efficacy Endpoints *36*
5.3 Imaging Scan Locations *36*
5.4 Assessment Time Points *37*
6. Safety Assessment **38**
6.1 Safety Indicators *38*
6.1.1 Routine Laboratory Safety Assessment Indicators 38
6.1.2 Physical Examination 38
6.1.3 12-Lead ECG 38
6.1.4 Vital Signs 39
6.1.5 Height and Weight 39
6.1.6 Pregnancy Test 39
6.1.7 Other Safety Checks 39
6.2 Safety Parameters *40*
6.3 Abnormal Laboratory Tests *40*

6.4 Safety Assessment Criteria ***40***

7. Adverse Events **40**

7.1 Adverse Event (AE) ***40***

7.2 AE Severity Grading Criteria ***41***

7.3 Serious Adverse Events (SAE) ***42***

7.4 Definition of Unexpected Safety Events ***43***

7.5 Collection and Follow-up of AEs/SAEs ***43***

7.6 Assessment of the Relationship Between AEs and the Study Drug ***44***

7.7 SAE Reporting Procedures ***45***

7.8 Unexpected Adverse Event Reporting ***45***

7.9 Pregnancy Report ***46***

7.10 Disease Progression and Death Reporting ***46***

8. Statistical Analyses **47**

8.1 Analysis Population ***47***

8.2 Statistical Methods ***48***

8.2.1 Subject Disposition 48

8.2.2 Demographic and Baseline Characteristics 48

8.2.3 Analysis of Primary Efficacy Endpoint 48

8.2.4 Analysis of Secondary Efficacy Endpoints 48

8.2.5 Safety Analyses 49

8.2.6 Compliance and Retention Analysis 50

9. Ethical Considerations **50**

9.1 Ethical Standards ***50***

9.2 Informed Consent ***51***

9.2.1 Informed Consent Form and Other Written Information Required by Subjects. 51

9.2.2 Informed Consent Process and Documentation 51

9.3 Confidentiality of Subject Information ***52***

10. Data Collection and Data Management **52**

10.1 Establishment of EDC Database ***52***

10.2 Data Entry and Verification ***52***

10.3 Data Review and Database Lock ***52***

11. Clinical Trial Quality Assurance **52**

12. References **54**

Study Flow Chart

	Screening Period		Treatment Period		End of Treatment	90-Day Safety Follow-up		Survival Follow-up Every 3 months
	-28-1 days	-7-1 days	Cycle 1 D1±3	Subsequent Cycles D1±3		30±7	30n±7 （n = 2, 3）	
Written Informed Consent[1]	X							
Inclusion/Exclusion Criteria	X							
Demographics/Smoking History/Past Medical History/Prior Medication[2]	X							
Sample Collection, Pathological Diagnosis and Genetic Testing[3]	X							
Vital Signs[4]	X		X	X	X	X		
Weight, Height[5]	X		X	X	X	X		
Physical Examination	X		X	X	X	X		
Concomitant Diseases	X							
ECOG Performance Status	X			X	X	X		
12-lead ECG[6]		X		X	X	X		
Complete Blood Count/Blood Biochemistry/Urinalysis/Stool Examination[7]		X		X	X	X		
Coagulation Function[8]		X		X	X	X		

Pregnancy Test		X			X			
Echocardiogram		X		Additional tests when clinically indicated.	X			
Myocardial Enzyme Panel[9]		X		Additional tests when clinically indicated.	X			
Thyroid Function[10]		X		Check once every cycle for cycles 2-4, and then once every 2 cycles thereafter.	X	X		
Hypothalamic-Pituitary-Adrenal (HPA) Axis Assessment[11]	X			Check once every cycle for cycles 2-4, and then once every 2 cycles thereafter.	X	X		
HIV, HBV, and HCV[12]	X							
Pulmonary Function Test[13]	X			Additional tests when clinically indicated.				
AE Assessment	X	X	X	X	X	X	X	
Concomitant Medications	X	X	X	X	X	X	X	
Vital Status						X	X	X
Subsequent Anti-tumor Therapy						X	X	X
Tumor Imaging Assessment[14]	X		Assessments will be conducted every 6 weeks (±7 days) for the first 12 months after the initial dose, and then every 12 weeks (±7 days) thereafter.		X	X*		
Quality of Life Score[15]		X		Performed at each imaging assessment	X			

Note:

1. Except for tumor imaging and tumor tissue biopsy that have already been performed within the specified time limit before the first dose, the signing of the ICF should be conducted before any protocol-specified procedures.
2. Prior medications include initial diagnostic treatment, including chemotherapy, radiotherapy, and surgery, etc. The date of the last anti-tumor treatment must be recorded.
3. **Sample Collection:** If the sample is from a core needle biopsy, it should be archived or freshly obtained within 6 months before the first dose. If it is a surgical specimen, the sample should be archived within 12 months before the first dose; **Pathological Diagnosis:** The pathological assessment of this trial will be conducted at each study site (on site review), and each trial center should archive the baseline pathological specimens. All pathological assessment results conducted study siteon site review during the screening period need to be secondarily confirmed by the leading unit of this project (Department of Pathology, Tongji Hospital Affiliated to Tongji Medical College

of Huazhong University of Science and Technology). Subjects need to have consistent results from both pathological assessments to be enrolled in this study. **Genetic Testing:** The study site must be able to provide relevant documents and materials for the subject's genetic testing. If the study site cannot provide these source files, the subject needs to be re-tested (NGS testing recommends that the subject provide at least 15 unstained slides and at least 2 mL of peripheral blood).

4. Vital signs include: body temperature, pulse, respiratory rate, and blood pressure. The investigator will measure the subject's blood pressure during the screening period and before each planned camrelizumab infusion.
5. Height measurement is only performed during the screening period. Weight measurement needs to be performed during the screening period, before each dose, at the end of treatment, and during safety follow-up.
6. 12-lead ECG examination time: during the screening period, before each camrelizumab administration, at the end of treatment, and during safety follow-up.
7. Complete blood count includes: red blood cell count (RBC), hemoglobin (HGB), hematocrit (HCT), white blood cell count (WBC), platelets (PLT), white blood cell differential [lymphocyte count (LYM), absolute neutrophil count (ANC), monocyte count (MONO), eosinophil count (EOS), basophil count (BASO)]. Blood biochemistry includes: liver function [total bilirubin (TBIL), alanine aminotransferase (ALT), aspartate aminotransferase (AST), γ-glutamyl transferase (γ-GT), alkaline phosphatase (ALP), albumin (ALB), total protein (TP), lactate dehydrogenase (LDH)]; renal function [blood urea nitrogen (BUN), urea (UREA), creatinine (Cr)]; blood electrolytes (Na, K, Cl, Mg, Ca, P). Urinalysis includes: acidity (PH), urine leukocytes (UALB), urine protein (UPRO), urine red blood cells (URBC), urine glucose (UGLU).
8. Coagulation function tests include: Thrombin Time (TT), prothrombin time (PT), activated partial thromboplastin time (APTT), and International Normalized Ratio (INR). These tests should be performed within 7 days prior to the first dose of study treatment, before camrelizumab administration on Day 1 of each cycle, at the end of treatment, and during safety follow-up.
9. Myocardial enzyme spectrum tests include: creatine kinase (CK) and creatine kinase-MB (CK-MB). Perform this test once within 7 days prior to the first dose of study treatment. Subsequently, this test should only be performed if symptoms such as precordial pain or palpitations occur, if there are electrocardiogram abnormalities, and at the end of treatment.
10. Thyroid function tests include: triiodothyronine (T3), thyroxine (T4), free triiodothyronine (FT3), free thyroxine (FT4), and thyroid stimulating hormone (TSH). These tests should be performed during the screening period, before camrelizumab administration on Day 1 of each cycle for cycles 2-4, before camrelizumab administration on Day 1 every 2 cycles thereafter, at the end of treatment, and during safety follow-up.
11. Hypothalamic-Pituitary-Adrenal (HPA) axis examination includes: corticotropin-releasing Hormone (CRH), adrenocorticotropic hormone (plasma ACTH), and adrenocortical hormone measurements. Adrenocortical hormone measurements include serum cortisol, urinary free cortisol (UFC), urinary 17-ketosteroids (17-KS), and urinary 17-hydroxycorticosteroids (17-KGS). Hospitals with the necessary resources are recommended to perform monitoring during the screening period, before camrelizumab administration on Day 1 of each cycle for cycles 2-4, before camrelizumab administration on Day 1 every 2 cycles thereafter, and at the end of treatment. (Hospitals with the necessary resources can perform this examination or some of these tests.)
12. This item includes HBV, HCV, and HIV antibody tests. HBV testing requirements: HBsAg should be tested during the screening period to determine HBV infection status. If positive, then HBsAg (quantitative), HBsAb (qualitative), HBcAb (qualitative), HBeAg (qualitative), HBeAb (qualitative), and HBV-DNA (qualitative, and quantitative if positive) should be tested. HCV testing requirements: HCV-Ab should be tested during the screening period to determine HCV infection status. If positive, then HCV-RNA (qualitative, and quantitative if positive) should be tested. If HBV carriers participate in the study, the investigator should arrange antiviral treatment as appropriate.
13. Pulmonary function tests include forced vital capacity (FVC), forced expiratory volume in 1 second (FEV1), and FEV1/FVC ratio. These tests should be performed during the screening period, and subsequently as determined by the investigator.
14. The method used for tumor imaging evaluation at baseline must be consistent with the method used for each subsequent follow-up assessment. Computed tomography (CT) or magnetic resonance imaging (MRI) scans are recommended. Based on the presenting signs and symptoms of each subject, examinations of other involved sites will be performed. Baseline assessments must include chest, upper abdomen (liver, gallbladder, pancreas, and spleen), brain, and all known or suspected sites of disease.

Subsequent clinical tumor imaging evaluations should include the chest, brain, and upper abdomen. Researchers may increase the frequency of imaging monitoring based on the actual situation. Baseline tumor assessments will be performed within 28 days prior to the first dose. Imaging data obtained before signing the informed consent can be used for screening tumor assessment, as long as it meets the protocol requirements. Imaging assessments will be conducted every 6 weeks (±7 days) within the first 12 months after the first dose, and every 12 weeks (±7 days) thereafter, until imaging-documented disease progression (PD) is recorded. For subjects with the first documented complete response (CR) or partial response (PR) and those with the first documented imaging-based PD, an additional imaging assessment should be performed 4 weeks (±7 days) later to confirm the response or PD. If PD is not confirmed, the subject will continue to receive study treatment and undergo imaging assessments until subsequent PD (while maintaining the imaging assessment frequency of every two cycles). Subjects with subsequent PD must discontinue treatment. X*: For subjects who discontinue treatment for reasons other than imaging-documented PD, imaging evaluations should be performed at the scheduled imaging assessment time points until any of the following events occur: initiation of new anti-tumor therapy, PD, subject withdrawal of informed consent (ICF), and/or death.

15. Quality of life score: Using the EOETC QLQ-C30 scale, completed by the investigator through communication with the subject. Administered once during the screening period, once at each imaging assessment, and once upon study withdrawal.

1. Study Background

1.1 Epidemiological Characteristics and Current Treatment Status of Pulmonary Sarcomatoid Carcinoma

Pulmonary sarcomatoid carcinoma (PSC) is a rare malignant lung tumor, accounting for 0.3% to 3% of all malignant lung tumors, with a generally higher prevalence in male smokers [1]. There are five subtypes: pleomorphic carcinoma, spindle cell carcinoma, giant cell carcinoma, carcinosarcoma, and pulmonary blastoma [2]. Current histological studies suggest that PSC is a transformative cancer originating from the same primitive epithelium, formed after epithelial-mesenchymal transition (EMT) [3].

PSC is poorly sensitive to chemoradiotherapy. A retrospective study showed that patients received a median of 3 cycles of first-line chemotherapy. The disease control rate (DCR) was only 31%, and the objective response rate (ORR) was only 16.5%. The median progression-free survival (PFS) was 2.0 months (95%CI: 1.8-2.3), and the median overall survival (OS) was only 6.3 months (95%CI: 4.7-7.8) [4]. The frequency of driver genes with potential for targeted therapy in PSC is much lower than in other types of NSCLC. TP53 may be the most common mutation type in PSC, followed by CDKN2B (23.2%), CDKN2A (37.6%), and NF1 (17.6%). Notably, the detected frequencies of EGFR mutation/BRAF (8.8%/7.3%) are both low [5]. Preliminary results of a study on volitinib in PSC patients with MET exon 14 skipping were presented as a poster at the 2020 American Society of Clinical Oncology (ASCO) Annual Meeting, with an ORR of 50%. However, patients carrying this mutation type account for only about 10%, and the vast majority of domestic PSC patients currently lack suitable targeted therapies or opportunities for targeted therapy.

1.2 Pulmonary Sarcomatoid Carcinoma and Immunotherapy

In recent years, immune checkpoint inhibitors, represented by programmed death 1 (PD-1) inhibitors and programmed death ligand 1 (PD-L1) inhibitors, have gradually emerged in the treatment of advanced NSCLC. In clinical studies, both immunotherapy alone and immunotherapy combined with chemotherapy can effectively improve the survival of NSCLC patients.

Studies have found that the PD-L1 expression level in PSC is significantly higher than in other types of NSCLC [6], and the positive rate of PD-L1 may be higher in Asian populations than in Caucasian populations [7]. In a Korean study, more than 90% of PSC patients were positive for PD-L1 expression [7]. In addition, approximately 43% of PSC patients exhibit a high tumor mutation burden (TMB) (defined as >10 mut/Mb) [5], and a high level of CD3+/CD163+ T cell infiltration has also been observed in tumor tissues in pathological sections [5, 6]. In a series of

case reports, among 5 PSC patients treated with PD-1 inhibitors, 1 achieved CR, 3 achieved PR, and 1 achieved SD, with an ORR of 80% and a DCR of 100% [8]. Another retrospective study reported that PSC patients treated with second-line immune checkpoint inhibitor monotherapy achieved an ORR of 40%, a median PFS of 4.89 months, and a median OS of more than one year [9]. Therefore, we believe that PSC may have a tumor immune microenvironment different from other NSCLCs, and this refractory pathological subtype, which is insensitive to traditional chemoradiotherapy and lacks effective driver genes, may be an advantageous population for immunotherapy.

1.3 Pulmonary Sarcomatoid Carcinoma and Anti-Angiogenic Therapy

Studies have reported that in pleomorphic carcinoma (the main pathological subtype of PSC), the expression of HIF-1α and VEGF is significantly higher than in other types of NSCLC patients [10]. In a case series report, 3 PSC patients were treated with apatinib combined with nab-paclitaxel and carboplatin; two patients achieved a best response of PR, and one patient maintained SD for more than 6 months [11]. In other types of sarcoma, oral multi-target anti-angiogenic TKIs are an important treatment modality. Whether it is sorafenib, sunitinib, pazopanib, anlotinib, or apatinib, significant efficacy has been achieved in various studies with soft tissue sarcoma and osteosarcoma as the main pathological subtypes. PSC has characteristics of both epithelial and mesenchymal tumors (sarcomas). It meets the indications for anti-angiogenic therapy, and theoretically, both epithelial and mesenchymal components can benefit from anti-angiogenic therapy.

1.4 Synergistic Effect of Anti-Angiogenic Therapy and Immunotherapy

The combination of anti-angiogenic therapy and immune checkpoint inhibitors has become an important anti-tumor strategy. Due to the overexpression of pro-angiogenic factors in tumor tissues, tumor vessels are usually disorganized, with excessively branched microvessels, and have stronger permeability compared to blood vessels in normal tissues. This abnormal vascular permeability is related to high interstitial fluid pressure and reduces blood perfusion and oxygenation. A reasonable dose of anti-angiogenic therapy can temporarily normalize tumor vessels by reducing vascular permeability and improving tumor perfusion and blood flow, alleviating the immunosuppressive state in the tumor microenvironment, thereby synergizing with immunotherapy. A phase III study (IMPOWER150) confirmed that adding bevacizumab (15 mg/kg) to cytotoxic chemotherapy (carboplatin, paclitaxel) and anti-PD-L1 antibody (atezolizumab) can prolong OS in NSCLC patients [12]. In another case report, nivolumab combined with anlotinib hydrochloride also achieved significant efficacy in the treatment of postoperative recurrent PSC [13].

In summary, patients with advanced PSC have poor sensitivity to traditional chemotherapy and limited accessibility to targeted therapy, and there is currently no standard first-line treatment. For this population, which potentially benefits from immunotherapy and anti-angiogenic therapy, whether combined immunotherapy and anti-angiogenic therapy can improve survival and thus be established as a standard treatment is the scientific question this study intends to address.

2. Study Drugs

2.1 Camrelizumab

Camrelizumab is a recombinant humanized anti-PD-1 monoclonal antibody that has been marketed in China and approved for relapsed or refractory classical Hodgkin's lymphoma after at least second-line systemic chemotherapy, advanced hepatocellular carcinoma previously treated with sorafenib and/or oxaliplatin-containing systemic chemotherapy, epidermal growth factor receptor (EGFR) gene mutation-negative and anaplastic lymphoma kinase (ALK)-negative, unresectable locally advanced or metastatic non-squamous non-small cell lung cancer as first-line treatment, and locally advanced or metastatic esophageal squamous cell carcinoma that has progressed or is intolerant after prior first-line chemotherapy. Similar drugs are already on the market abroad. Preclinical studies have shown that camrelizumab has good anti-tumor activity. Phase I clinical trials in multiple tumor types have preliminarily validated the safety, tolerability, and efficacy of camrelizumab monotherapy in advanced solid tumors.

2.2 Famitinib Malate Capsules

Famitinib (code name: SHR1020) is a small molecule multi-target tyrosine kinase inhibitor that significantly inhibits kinases such as c-Kit, VEGFR1/2/3, PDGFRα/β, FGFR2/3, Ret, Axl, Mer, Flt3, and Fms.

The pharmacokinetic results of the famitinib phase I clinical study (study number: FMTN-I-1.1, HR-FMTN-I-PK) showed that after a single oral dose of famitinib in patients with advanced solid tumors, the time to peak concentration of famitinib was approximately 3-6 h, and it was widely distributed in the body and eliminated slowly, with an elimination half-life of approximately 41-45 h. The time to peak concentration of the N-deethylated metabolite of famitinib in the body was similar to that of the parent drug, but the elimination in the body was slower than that of the parent drug, and its plasma exposure was less than 10% of the parent drug. After continuous QD administration of apatinib, the plasma trough concentrations of the parent drug and the N-deethylated metabolite reached steady state at approximately 7-10 days and 10-14 days, respectively. At steady state, both the parent drug and the N-deethylated

metabolite accumulated in the body, and the accumulation of the metabolite was more pronounced. In the dose range of 4-27 mg, the C_{max} and AUC_{0-24} of famitinib increased with increasing dose.

Based on existing clinical data for famitinib, from four clinical trials available for statistical analysis (FMTN-I-PK, HR-FMTN-IId-III-CRC, FMTN-II-203-NSCLC, and SHR-1210-II213), the pooled incidence of adverse drug reactions (ADRs) occurring in ≥10% of patients in famitinib monotherapy studies (N=393) included white blood cell count decreased (52.4%), neutrophil count decreased (50.9%), platelet count decreased (46.6%), palmar-plantar erythrodysesthesia syndrome (39.9%), hypertension (36.4%), proteinuria (35.1%), blood pressure elevated (24.2%), presence of urine protein (21.6%), aspartate aminotransferase increased (16.3%), fatigue (14.2%), bilirubin increased and diarrhea (13.7% each), alanine aminotransferase increased (13%), thyroid stimulating hormone increased (12.5%), hemoglobin decreased (10.9%), decreased appetite (10.7%), and anemia (10.2%).

The pooled incidence of famitinib-related adverse events (AEs) occurring in ≥10% of patients in famitinib combination therapy studies (N=152) included hypertension (55.9%), neutrophil count decreased (50%), white blood cell count decreased (48%), platelet count decreased (47.4%), palmar-plantar erythrodysesthesia syndrome (44.7%), proteinuria (43.4%), anemia (32.2%), aspartate aminotransferase increased (25.7%), alanine aminotransferase increased (23.7%), hypertriglyceridemia (23%), hypercholesterolemia (21.1%), gamma-glutamyl transferase increased (20.4%), diarrhea (19.7%), fatigue (15.1%), serum creatinine increased (14.5%), urinary tract infection (13.8%), hypothyroidism (13.2%), decreased appetite and hematuria (11.8% each), serum alkaline phosphatase increased and hyperuricemia (11.2% each), and weight loss (10.5%).

2.3 Rationale for the Famitinib Malate Capsules Dosage Regimen Design in the Combination Therapy

In a Phase II study initiated by Hengrui Pharmaceuticals, "A Study of Funitinib Malate Combined with camrelizumab for the Treatment of Advanced Urinary System and Gynecological Tumors" (SHR-1210-II-213, NCT03827837), the tolerability of famitinib 20 mg qd in combination with SHR-1210 was observed in the first 12 enrolled subjects for 2 cycles (6 weeks). A summary of famitinib dose adjustments for all 49 enrolled subjects up to the completion of the tolerability observation (June 30, 2019) showed good tolerability. Only one subject interrupted famitinib for more than 14 days due to thyroiditis during the tolerability observation period (determined as DLT); only one subject out of all 49 subjects had their famitinib dose reduced due to diarrhea. Based on the incidence of clinically significant toxicities

and the overall dose adjustment status at the end of the tolerability observation period, 20 mg qd of famitinib was determined as the recommended dose for the combination therapy. Furthermore, in the cervical cancer cohort, among 33 subjects with recurrent and metastatic disease who had received ≥1 line of prior therapy, 30 subjects were evaluable. The evaluation results (best overall response) were 1 CR, 14 PR, 9 SD, and 6 PD, with a response rate of 50.0% and a disease control rate of 80.8%. In the renal cancer cohort, among 38 subjects with metastatic renal cell carcinoma (25 of whom had received ≥1 line of prior therapy), 36 were evaluable. The evaluation results were 23 PR, 11 SD, and 2 PD, with a response rate of 63.9% and a DCR of 94.4%. This confirms the prominent preliminary efficacy of camrelizumab 200 mg q3w combined with famitinib malate 20 mg qd. Therefore, SHR-1210 200 mg fixed dose, q3w combined with famitinib 20 mg, qd continuous oral administration was selected as the combination therapy.

2.4 Risk/Benefit Assessment

2.4.1 Main Safety Risks

Observed Adverse Reactions of Camrelizumab:

In subjects treated with camrelizumab, the very common and common adverse reactions possibly related to camrelizumab include the following:

Very common (incidence ≥1/10): Reactive cutaneous capillary proliferation, various rashes (including macular rash, maculopapular rash, papular rash, pruritic rash, erythematous rash, pustular rash), hypothyroidism, fatigue, elevated transaminases (AST, ALT).

Common (incidence ≥1/100, <1/10): Pruritus, fever, proteinuria, cough, pneumonia, hyperthyroidism, diarrhea, nausea, decreased appetite, musculoskeletal pain, anemia, leukopenia, neutropenia, thrombocytopenia, blood bilirubin increased, γ-glutamyl transferase increased, blood alkaline phosphatase increased.

The vast majority of the above adverse reactions are Grade 1-2 and can be controlled with or without medical intervention. Reactive cutaneous capillary proliferation is a unique skin reaction to SHR-1210, with a higher incidence in SHR-1210 monotherapy, but the severity is mild, clinically tolerable, and reversible after drug discontinuation. In other clinical trials of SHR-1210 combined with famitinib, the incidence of reactive cutaneous capillary proliferation has shown a significant downward trend.

In subjects treated with camrelizumab, the common and uncommon serious adverse reactions observed include the following:

Common (incidence ≥1/100, <1/10): Pneumonia, liver dysfunction, pulmonary infection, thrombocytopenia.

Uncommon (incidence ≥1/1000, <1/100): Ulcerative colitis, diarrhea, reactive capillary proliferation (skin, oral, nasal), fatigue, fever, infusion-related reactions, decreased appetite, hypothyroidism, anemia, leukopenia, neutropenia, upper respiratory tract infection.

Since PD-1 is a molecule that modulates immune function by turning off the immune system, and antibodies against PD-1 can prevent the shutdown of the immune system, potential adverse reactions may be related to a potentially enhanced immune response. Immune-related reactions observed with camrelizumab include: immune-related pneumonitis and interstitial lung disease, hepatitis, colitis, myocarditis, myositis, thyroiditis, hypopituitarism, adrenal insufficiency. Nephritis and uveitis have also been observed with other PD-1 antibodies. These are all class effects of immune-mediated drugs and require special attention. Most of these immune-related adverse reactions are mild and controllable, while a few are serious adverse events (SAEs) and may even be life-threatening.

For all large molecule protein monoclonal antibody drugs, other risks also include infusion reactions: mainly chills, shivering, cyanosis of the face and extremities, followed by fever, which may be accompanied by nausea, vomiting, headache, dizziness, restlessness, delirium, etc. In severe cases, there may be coma, decreased blood pressure, shock, and respiratory failure. These are caused by a variety of factors during intravenous infusion, such as pyrogens, drugs, impurities, excessively low drug solution temperature, excessively high drug solution concentration, and excessively fast infusion rate. Camrelizumab, as a fully humanized monoclonal antibody, has been reported to have a low incidence of infusion reactions, and the severity is also relatively mild.

Other serious, even life-threatening or fatal adverse reactions observed in similar PD-1 antibodies include: hypersensitivity reactions, encephalitis, myocarditis, polymyositis, rhabdomyolysis, toxic epidermal necrolysis, Stevens-Johnson syndrome, renal failure.

Known Adverse Reactions of Famitinib:

According to the existing famitinib clinical data, common drug-related adverse events include neutrophil count decreased, white blood cell count decreased, platelet count decreased, anemia/hemoglobin decreased, red blood cell count decreased; proteinuria, protein detected in urine, hypertension, blood pressure increased, elevated transaminases, elevated alanine aminotransferase, elevated aspartate aminotransferase, γ-glutamyl transferase increased, blood alkaline phosphatase increased, blood bilirubin increased, blood cholesterol increased, blood triglycerides abnormal, blood triglycerides increased, hyperlipidemia, thyroid stimulating hormone increased, hypothyroidism, blood creatinine increased, blood urea increased, glucose detected in urine, positive urine red blood cells, palmar-plantar erythrodysesthesia syndrome,

rash, radiation dermatitis, nausea, vomiting, oropharyngeal pain, oral ulcer, oral mucositis, decreased appetite, oropharyngeal pain, fatigue, diarrhea, epistaxis, hemoptysis, hematemesis, hematochezia, pain, headache, abdominal pain, venous thrombosis, peripheral edema, head and facial edema.

Most of the above adverse reactions can be recovered and relieved after drug interruption and treatment. In addition, when using famitinib, special attention should be paid to some events with low incidence but potentially serious consequences, even death, including various types of bleeding (intracranial hemorrhage, gastrointestinal bleeding, hematuria), thrombotic events (cerebral infarction, myocardial infarction), cardiotoxicity (QT interval prolongation, arrhythmia), and hepatotoxicity.

Observed Adverse Reactions of Camrelizumab Combined with Famitinib

In an open-label, multi-center Phase II clinical study of camrelizumab combined with famitinib for the treatment of advanced urological and gynecological tumors, a total of 160 subjects were enrolled as of October 31, 2020. The first subject was enrolled on January 23, 2019, and the 160th subject was enrolled on October 30, 2020, with a median follow-up time of 12.9 months. The most common drug-related AEs (incidence ≥10%) with camrelizumab combined with famitinib were hematological, including platelet count decreased,neutrophil count decreased, white blood cell count decreased, and anemia, and non-hematological, including proteinuria, hypertension, palmar-plantar erythrodysesthesia syndrome, diarrhea, ALT increased, AST increased, hypertriglyceridemia, elevated GGT, hypercholesterolemia, hypothyroidism, fatigue, blood creatinine increased, hyperuricemia, decreased appetite, urinary tract infection, weight loss, blood alkaline phosphatase increased, hyperglycemia, hematuria, and rash.

Grade 3 or higher drug-related AEs with an incidence ≥2% were hematological, including neutrophil count decreased, platelet count decreased, anemia, white blood cell count decreased, and lymphocyte count decreased, and non-hematological, including hypertension, palmar-plantar erythrodysesthesia syndrome, hypertriglyceridemia, proteinuria, GGT increased, AST increased, and ALT increased.

The incidence of immune-related adverse events (irAEs) was 28.1%, mostly Grade 1-2, which were relieved or resolved after interruption of the study drug and corrective treatment. The incidence of irAEs ≥ Grade 3 was 4.4%, including: reactive angioendotheliomatosis in 1 subject (0.6%), diarrhea 1 in subject (0.6%), enteritis in 1 subject (0.6%), oral ulcer (0.6%), rash (0.6%), drug eruption (0.6%), GGT increased in 1 subject (0.6%), conjugated bilirubin increased in 1 subject (0.6%), AST increased in 1 subject (0.6%), serum bilirubin increased in 1 subject (0.6%), and proteinuria increased in 1 subject (0.6%).

A total of 33 subjects (20.6%) experienced at least one drug-related SAE. Among them, 27 subjects (16.9%) experienced at least one Grade ≥3 drug-related SAE. SAEs related to the study drug included platelet count decreased in 6 subjects (3.8%), anemia in 5 subjects (3.1%), and vaginal bleeding in 3 subjects (1.9%).

2.4.2 Potential Benefits

Currently, there is no standard treatment for PSC. For the population enrolled in this study - PSC without driver gene mutations - the main clinical treatment is platinum-based chemotherapy, which has limited efficacy. The treatment regimen in this study, the anti-PD-1 antibody camrelizumab combined with the multi-target anti-angiogenic drug famitinib malate, has shown promising preliminary efficacy in various tumors (including non-small cell lung cancer). Its efficacy is characterized by improving the response rate of PD-1 monotherapy while retaining the long-lasting efficacy and survival benefits of PD-1 monotherapy. By participating in this study and receiving the study drug, subjects may benefit from:

1) Higher tumor response rate, higher tumor disease control rate;

2) Sustained duration of response, or continued disease stability. This ultimately translates into longer PFS and OS.

At the same time, by participating in this study, subjects can receive more comprehensive diagnosis and treatment, including comprehensive disease staging, pathological typing, molecular diagnosis, physical examinations, comprehensive examination assessment, and recommendations for optional treatment plans.

3. Study Objectives and Endpoints

3.1 Study Objectives

Primary Objective: To evaluate the ORR of camrelizumab combined with famitinib malate capsules as first-line treatment for locally advanced or metastatic pulmonary sarcomatoid carcinoma;

Secondary Objectives:

1) To evaluate the DCR, DoR, PFS, and OS of camrelizumab combined with famitinib malate capsules as first-line treatment for locally advanced or metastatic pulmonary sarcomatoid carcinoma; to evaluate changes in disease-related symptoms in each group of subjects based on the quality of life score (EOETC QLQ-C30) table;
2) To evaluate the safety of camrelizumab combined with famitinib malate capsules as first-line treatment for locally advanced or metastatic pulmonary sarcomatoid carcinoma;

1) **Exploratory Objective：** To explore the potential genomic characteristics of the pulmonary sarcomatoid carcinoma population;

3.2 Study Endpoints

3.2.1 Primary Endpoint

Objective Response Rate (ORR) as defined by RECIST v1.1;

3.2.2 Secondary Endpoints

1. Disease Control Rate (DCR), Progression-Free Survival (PFS), Overall Survival (OS), Duration of Response (DoR) and Quality of life score;
2. The safety of camrelizumab combined with famitinib malate capsules as first-line treatment for locally advanced or metastatic pulmonary sarcomatoid carcinoma;

3.2.3 Exploratory Endpoint：

1. Subjects' genomic data and expression of immune microenvironment-related biomarkers

4. Study Design

This is a prospective, multi-center, open-label, single-arm clinical study designed to evaluate the efficacy and safety of camrelizumab combined with famitinib malate capsules in the treatment of locally advanced or metastatic pulmonary sarcomatoid carcinoma. Each subject will be treated until disease progression or intolerable drug-related toxicity, unless the subject actively withdraws from the trial or the investigator deems the subject unsuitable for further trials. Subsequently, the subject will enter the survival follow-up period.

4.1 Study Design Diagram

Because this study involves a relatively small population and lacks corresponding first-line standard treatment, the purpose of the study is to explore the preliminary efficacy and safety of this combination therapy in the target population on a small scale, therefore, a single-arm study design is adopted.

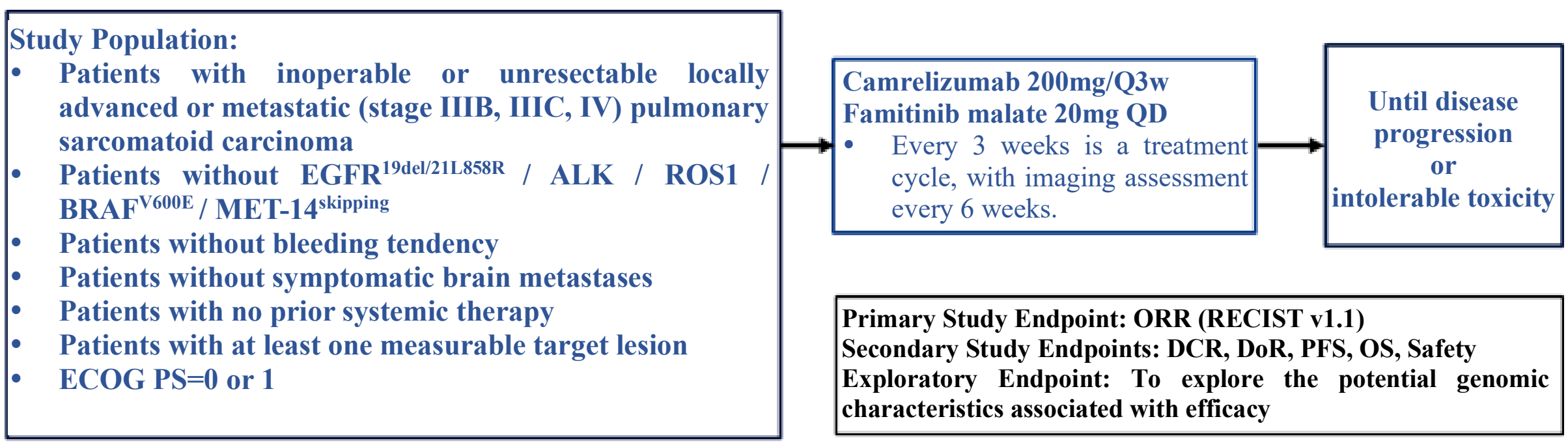

***Subjects are required to provide sufficient samples for PD-L1 immunohistochemical staining and NGS testing before enrollment.**

4.2 Study Population

4.2.1 Sample Size Determination

This study adopts a Simon's two-stage design, with the primary endpoint being ORR. Based on previous research data[4, 14], it is estimated that this study can achieve an ORR of 35%. If the ORR is less than 15%, the efficacy is considered poor. Assuming a one-sided type-I-error of 5% and statistical power of 80%, in the first stage it was required to have 15 patients and if at least 3 patients achieved CR or PR as per RECIST v1.1, the study would proceed to the second stage and continue to enroll another 13 evaluable patients. In total, 28 evaluable patients were required. To establish a significant result, it was required to have at least 8 patients achieving CR or PR out of 28 evaluable patients at the end of the second stage.

4.2.2 Patient Selection

Inclusion Criteria	Patients must meet the following criteria to be enrolled in this study:<br>1. Age ≥18 years and ≤75 years;<br>2. Patients with histologically confirmed unresectable, untreatable with radical chemoradiotherapy stage IIIB, IIIC, and IV pulmonary sarcomatoid carcinoma (according to the WHO pulmonary sarcomatoid carcinoma criteria, the pathological specimen contains spindle and/or giant cells, pleomorphic carcinoma, giant cell carcinoma, carcinosarcoma, or blastoma components). Patients are also eligible if the biopsy specimen is diagnosed as non-small cell lung cancer (NSCLC) with sarcomatoid components (sarcomatoid component tumor cells can be spindle cells, and/or giant cells, and/or heterologous sarcoma differentiation, including rhabdomyosarcoma, chondrosarcoma, etc.). [Example: diagnosed as non-small cell lung cancer, not otherwise specified (NSCLC-NOS), with spindle cell component].<br>3. Patients with no prior systemic therapy for advanced/metastatic NSCLC; chemotherapy and/or radiotherapy are allowed as part of neoadjuvant/adjuvant therapy, and patients can be enrolled if ≥6 months have elapsed since the end of treatment until recurrence or metastasis;<br>4. Patients with Eastern Cooperative Oncology Group (ECOG) performance status of 0 or 1;<br>5. According to RECIST 1.1 criteria, patients must have at least one measurable lesion (lesions that have received radiotherapy must show clear progression before they can be considered measurable lesions);<br>6. Sufficient tumor tissue samples (fresh or paraffin-embedded) obtained at or after the diagnosis of advanced or metastatic disease and prior to any radiotherapy must be provided for biomarker exploration (including but not limited to PDL1 immunohistochemical staining and NGS testing). If the sample is from a core needle biopsy, it must be archived or freshly obtained within 6 months prior to the first dose. If the sample is a surgical specimen, it must be archived within 12 months prior to the first dose.<br>7. Life expectancy of more than 3 months;<br>8. Adequate bone marrow function, defined as absolute neutrophil count ≥1.5×10^9/L, platelet count ≥100×10^9/L, and hemoglobin ≥90 g/L [without blood transfusion or erythropoietin (EPO) dependency within 7 days];<br>9. Good liver function, defined as total bilirubin level ≤1.5 times the upper limit of normal (ULN); AST and ALT levels ≤2.5 times ULN for patients without liver metastases; AST and ALT levels ≤5 times ULN for patients with documented liver metastases;<br>10. Adequate renal function, defined as serum creatinine ≤1.5 times theULN or a calculated creatinine clearance ≥60 mL/min (Cockcroft-Gault formula); urinalysis showing less than 2+ proteinuria. If the patient has ≥2+ proteinuria at baseline, a

	24-hour urine collection should be performed and demonstrate a 24-hour urine protein quantification ≤1 g;<br>11. Adequate coagulation function, defined as an international normalized ratio (INR) or prothrombin time (PT) ≤1.5 times the ULN; if the subject is receiving anticoagulant therapy, as long as the PT is within the intended therapeutic range of the anticoagulant.<br>12. For female subjects of childbearing potential, a serum pregnancy test must be performed within seven days prior to the first dose of study drug (Cycle 1, Day 1), and the result must be negative. Subjects must agree to use effective contraception during the study and for 90 days after the last dose of study drug, or be surgically sterilized.<br>13. Able to comply with the study and follow-up procedures;<br>14. Written informed consent must be obtained before any trial-related procedures are implemented.
Exclusion Criteria	Patients will be excluded from enrollment if they have any of the following conditions:<br>1. Imaging (CT or MRI) confirms tumor invasion of major blood vessels or unclear boundaries with blood vessels; within 3 months prior to screening, a single episode of hemoptysis ≥2.5 mL;<br>2. Patients with EGFR sensitizing mutations (19 Exon del/L858R), ALK, ROS1 gene rearrangements or fusions, $BRAF^{v600e}$ mutations, or MET exon 14 skipping mutations;<br>3. Patients with active bleeding or perforation or the presence of hereditary or acquired bleeding diathesis;<br>4. Patients with hypertension whose blood pressure cannot be controlled within the normal range (systolic blood pressure ≤140 mmHg/diastolic blood pressure ≤90 mmHg) with antihypertensive medication;<br>5. Urine routine test indicates urinary protein ≥(++), and 24-hour urinary protein quantification is ≥1.0 g;<br>6. Presence of thrombotic diseases requiring long-term anticoagulation therapy with warfarin or heparin, or requiring long-term antiplatelet therapy (aspirin ≥300 mg/day or clopidogrel ≥75 mg/day);<br>7. There are multiple factors that can affect the absorption of oral medications, such as inability to swallow, nausea and vomiting, chronic diarrhea, and intestinal obstruction.<br>8. Symptomatic, untreated, or actively progressing central nervous system (CNS) metastases are confirmed by CT or MRI assessment during screening and prior to radiological imaging assessment. However, asymptomatic patients with stable and treated CNS lesions are also eligible if all of the following criteria are met:<br>- Measurable lesions, as determined by RECIST v1.1, must exist outside the CNS;<br>- The patient has no history of intracranial hemorrhage or spinal cord hemorrhage;<br>- The patient has not undergone stereotactic radiotherapy within 7 days prior to the start of study treatment, whole-brain radiotherapy within 14 days prior to the start of study treatment, or neurosurgical resection within 28 days prior to the start of study treatment;<br>- Imaging confirmed stability for at least 4 weeks before enrollment, and systemic corticosteroid therapy (dose ≤10mg/day prednisone or other equivalent corticosteroids) has been discontinued for more than 2 weeks;<br>- Metastasis is limited to the cerebellum or supratentorial region (i.e., no metastasis to the midbrain, pons, medulla oblongata, or spinal cord);<br>9. Patients are currently participating in interventional clinical research therapy, or having received other investigational drugs or used investigational devices within 4 weeks prior to the first dose;<br>10. Received Solid organ or hematopoietic stem cell transplantation;<br>11. Patients had active autoimmune disease that required systemic therapy (e.g., with disease-modifying agents, corticosteroids, or immunosuppressants) within 2 years before the first dose. Replacement therapy (e.g., thyroxine, insulin, or physiologic corticosteroid replacement for adrenal or pituitary insufficiency) is not considered as systemic therapy.<br>12. Diagnosed with immunodeficiency or receiving systemic glucocorticoid

	therapy or any other form of immunosuppressive therapy within 7 days prior to the first dose of the study drug; physiological doses of glucocorticoids (≤10 mg/day of prednisone or equivalent) are permitted.<br>13. History of non-infectious pneumonitis requiring corticosteroid treatment within 1 year prior to the first dose or current interstitial lung disease;<br>14. Patients with myocardial ischemia or myocardial infarction of grade II or higher, or uncontrolled arrhythmia (including QTc interval ≥450 ms for males, ≥470 ms for females). Patients with NYHA class III-IV heart failure or echocardiography: LVEF (left ventricular ejection fraction) <50%;<br>15. Patients have a known history of human immunodeficiency virus (HIV) infection;<br>16. Patients with untreated active hepatitis B;<br>Note: Patients with hepatitis B who meet the following criteria may also be included:<br>a. HBV viral load must be <1000 copies/mL (200 IU/mL) before the first dose. Patients should receive anti-HBV therapy throughout the study drug treatment period to prevent viral reactivation.<br>b. For patients who are anti-HBc positive (+), HBsAg negative (-), anti-HBs negative (-), and have a negative (-) HBV viral load, prophylactic anti-HBV treatment is not required, but close monitoring for viral reactivation is necessary.<br>17. Patients with active HCV infection (positive HCV antibody and HCV-RNA levels above the detection limit);<br>18. Patients have experienced serious infections within 4 weeks prior to the first dose, including but not limited to infectious complications requiring hospitalization, bacteremia, and severe pneumonia; patients with any active infection are excluded; the possibility of lymph node metastasis of lung cancer is not excluded;<br>19. Pregnant and breastfeeding women;<br>20. Known allergy to camrelizumab, famitinib malate capsules, or any of their excipients;<br>21. Patients with malignant tumors other than NSCLC within 5 years before enrollment are eligible, except for adequately treated carcinoma in situ of the cervix, basal cell or squamous cell skin cancer, localized prostate cancer after radical surgery, and ductal carcinoma in situ after radical surgery.<br>22. The investigator judges that the patients have other factors that may lead to premature termination of this study.

4.2.3 Subject Withdrawal Criteria

1. The subject requests to withdraw from the treatment or withdraws informed consent;
2. Disease progression confirmed by imaging examinations;
3. Poor subject compliance;
4. The subject cannot tolerate toxicity;
5. The subject is lost to follow-up, dies, or becomes pregnant;
6. Other situations where the investigator deems it necessary to discontinue the treatment.

4.2.4 Study Termination Criteria

1) Unexpected, significant, or unacceptable risks to subjects are discovered;
2) The study drug/investigational treatment is ineffective, or continued testing is meaningless;
3) The sponsor decides to terminate the study due to reasons such as serious delays in subject enrollment or frequent protocol deviations.

4.3 Treatment Regimen and Dosage

4.3.1 Treatment Regimen

[Screening Period]: Subjects must undergo screening evaluations within 28 days prior to the first dose to determine their eligibility for the study. Tumor specimens need to be collected during the screening period (archived within the past 6 months or freshly obtained tumor tissue blocks or pathological biopsy sections; if it is a surgical specimen, the sample should be archived within 12 months prior to the first dose).

[Treatment Period]: Eligible subjects will begin treatment within 3 days of enrollment.

Camrelizumab: Intravenous infusion, fixed dose of 200 mg, administered as a 30-minute intravenous infusion (including flushing time, the total infusion time should not be less than 20 minutes and not more than 60 minutes), once every 3 weeks. Until disease progression and/or occurrence of intolerable toxic reactions;

Famitinib Malate Capsules: 20 mg, administered orally once daily, before meals (dosing time should be as consistent as possible each day), taken with lukewarm water.

Each treatment cycle is 3 weeks. Drug treatment is administered on Day 1 of each cycle. The administration time window can be ±3 days. But within 72 hours before each administration, except for imaging examinations, the subjects must complete all the examinations stipulated in the protocol (including vital signs, physical examination, laboratory tests, performance status score, and other examinations specified in the study protocol). Subjects also need to complete clinical tumor imaging evaluations (using consistent evaluation methods, either CT or MRI) every 6 weeks (±7 days) within 12 months after the first dose, and every 12 weeks (±7 days) after 12 months from the first dose.

When a subject discontinues treatment or withdraws from the study, a complete examination is still required, including vital signs, physical examinations, laboratory tests, quality of life assessment, clinical tumor imaging evaluation (CT or MRI), etc.

[Follow-up Period]: After subjects are withdrawn from the study, safety follow-up is required. Subjects will be followed up every 30±7 days for up to 90 days. The first safety visit (30±7 days after withdrawal) must be conducted at the study site, and vital signs, physical examination, laboratory tests, etc. should be performed to assess AEs, concomitant medications, and concomitant therapies. The other two visits (60±7 days and 90±7 days after withdrawal) are telephone visits, and only survival information, AEs, concomitant medications, and concomitant therapies need to be collected.

After the safety follow-up, subjects will be followed up every 3 months for survival status via clinic or telephone visits until death, loss to follow-up, withdrawal of informed consent,

completion of 2 years of observation, or termination of the study.

4.3.2 Dosage Regimen

Camrelizumab is administered at a fixed dose of 200 mg on Day 1 of each cycle. The dosing window can be ±3 days, but within 72 hours before each dose, except for imaging examinations, the subject must complete all clinically required examinations to assess the tolerability of continued medication. Subjects are advised to remain hospitalized for observation for 72 hours after the first dose. Camrelizumab is administered via intravenous infusion over 30 minutes (no less than 20 minutes and no more than 60 minutes, including the flushing phase). The intravenous infusion should be administered through a medical infusion bag with an in-line filter (0.2 μM). Other medications should not be administered through the same intravenous line before or after the infusion.

20 mg of famitinib capsules are administered orally on Day 1 of each cycle, once daily, before meals (the time of daily dosing should be as consistent as possible) until disease progression. The dosing window can be ±3 days. But within 72 hours before each dose, except for imaging examinations, subjects must complete all clinically required examinations to assess the tolerability of continued medication.

4.4 Dose Adjustment and Discontinuation

4.4.1 General Principles of Dose Adjustment

1) The reasons for dose adjustment or treatment delay, the measures taken, and the results should be recorded in the subject’s medical records and electronic case report form (CRF);
2) If concomitant symptoms are present at baseline, the investigator will determine whether to adjust the dose according to the change in the grade of adverse reactions. For example, if the subject’s “asthenia” status is graded as Grade 1 at baseline, and during the study treatment, the “asthenia” status is graded as Grade 2, this means that the subject’s “asthenia” status has increased by one grade, and the dose should be adjusted according to Grade 1 toxicity;
3) If several adverse reactions of different grades or severity occur simultaneously, dose adjustments will be based on the highest grade observed;
4) If dose adjustment is required solely due to abnormal hematological laboratory values, the dose will be adjusted based on the hematological test values before the start of the treatment cycle;
5) If, in the investigator’s judgment, the adverse reaction is unlikely to further develop into a serious or life-threatening event, the current dose will be continued without dose adjustment or treatment interruption.

6) If, in the investigator's judgment, the toxicity is caused by one of the study drugs, the dose of the other drug(s) does not need to be adjusted;
7) Permanent discontinuation of one study drug before the occurrence of PD will not affect the continued use of other study drugs;
8) If the treatment with one study drug is interrupted due to adverse reactions to that drug, but not all drugs are interrupted, this treatment is still considered as one treatment cycle.

4.4.2 Camrelizumab Dose Adjustment

Increasing or decreasing the dose of camrelizumab is not allowed, only interruption or discontinuation is permitted. The maximum allowed interruption period for camrelizumab is 9 weeks (calculated from the actual administration time of the last dose).

The camrelizumab dose adjustment guidelines are shown in Table 1.

In addition, for PD assessed by the investigator according to RECIST 1.1 criteria, the drug should be permanently discontinued unless the subject meets the criteria for continued treatment after progression. Subjects are allowed to interrupt medication for other medical or surgical procedures, or accidents (holidays) unrelated to the study treatment. Subjects should resume study treatment within 2 weeks after the interruption of treatment (the planned administration time), unless there are other resolutions based on the discussion of the investigator and the sponsor. The reason for treatment interruption must be recorded in the CRF.

Table 1. Camrelizumab Dose Adjustment Guidelines

	Severity+	Treatment Adjustment Plan
Reactive capillary hemangiomatosis*	Grade 3	The treatment should be interrupted until the adverse reaction recovers to Grade 0-1.
	Grade 4	The treatment should be permanently discontinued
Immune-Related Adverse Events		
Pneumonia	Grade 2	The treatment should be interrupted until the adverse reaction recovers to Grade 0-1.
	Grade 3 or 4 or Recurrent Grade 2	The treatment should be permanently discontinued
Diarrhea and colitis	Grade 2 or 3	The treatment should be interrupted until the adverse reaction recovers to Grade 0-1.
	Grade 4	The treatment should be permanently discontinued
Hepatitis (applicable to non-hepatocellular carcinoma patients)	Grade 2, AST, ALT at 3-5 times the ULN or total bilirubin (TBIL) at 1.5-3 times ULN	The treatment should be interrupted until the adverse reaction recovers to Grade 0-1.
	Grade 3 or 4, AST, ALT >5 times ULN, or total bilirubin >3 times ULN	The treatment should be permanently discontinued
Hepatitis (applicable to patients with	AST or ALT: 3-5 times ULN, if baseline status is within normal range	The treatment should be interrupted until the adverse reaction recovers to

hepatocellular carcinoma)	AST or ALT: 5-10 times ULN. If baseline AST or ALT is between 1-3 times ULN	Grade 0-1 or returns to baseline, then resume dosing.
	AST or ALT: 8-10 times ULN, if baseline status is 3-5 times ULN<br>Total bilirubin: 1.5-3 times ULN	
	AST or ALT: >5 times ULN, if baseline status is within normal range<br>AST or ALT: >10 times ULN, if baseline status >ULN<br>Total bilirubin > 3 times ULN	The treatment should be permanently discontinued
Nephritis	Grade 2 or 3 Serum Creatinine Elevation	The treatment should be interrupted until the adverse reaction recovers to grade 0-1.
	Grade 4 blood creatinine increased	The treatment should be permanently discontinued
Endocrine diseases	Symptomatic Grade 2 or 3 hypothyroidism, Grade 2 or 3 hyperthyroidism, Grade 2 or 3 hypophysitis, Grade 2 adrenal insufficiency<br>Grade 3 hyperglycemia or diabetes mellitus	The treatment should be interrupted until the adverse reaction recovers to grade 0-1.
	Grade 4 hypothyroidism<br>Grade 4 hyperthyroidism<br>Grade 4 hypophysitis<br>Grade 3 or 4 adrenal insufficiency<br>Grade 4 hyperglycemia or diabetes mellitus	The treatment should be permanently discontinued
Skin adverse reactions	Grade 3	The treatment should be interrupted until the adverse reaction recovers to Grade 0-1.
	Grade 4, Stevens-Johnson syndrome (SJS) or toxic epidermal necrolysis (TEN)	The treatment should be permanently discontinued
Thrombocytopenia	Grade 3	The treatment should be interrupted until the adverse reaction recovers to Grade 0-1.
	Grade 4	The treatment should be permanently discontinued
Other irAEs	Grade 3 or 4 blood amylase increased or lipase increased<br>Grade 2 or 3 pancreatitis<br>Grade 2 myocarditis<br>Other first-occurrence irAEs of Grade 2 or 3	The treatment should be interrupted until the adverse reaction recovers to Grade 0-1.
	Grade 4 pancreatitis or recurrent pancreatitis of any grade<br>Grade 3 or 4 myocarditis<br>Grade 3 or 4 encephalitis<br>Other irAEs first occurring at Grade 4	The treatment should be permanently discontinued
Recurrent or persistent adverse reactions	Recurrent Grade 3 or 4 (excluding endocrine disorders)<br>Grade 2 or 3 AEs that do not improve to Grade 0-1 within 12 weeks after the last dose (excluding endocrine disorders)<br>Within 12 weeks after the last dose,	The treatment should be permanently discontinued

	the corticosteroid dose could not be reduced to ≤10 mg/day prednisone equivalent.	
Infusion reactions	Grade 2	The infusion rate should be reduced or the treatment should be interrupted. After symptoms resolve, resumption of treatment may be considered with close observation.
	Grade 3 or 4	The treatment should be permanently discontinued

+Adverse reaction severity is based on the National Cancer Institute's Common Terminology Criteria for Adverse Events, version 4.03 (NCI-CTCAE v4.03).
*Reactive capillary hemangiomas are graded according to the "CSCO Guidelines for the Management of Immune Checkpoint Inhibitor-Related Toxicity 2019".
**The safety of restarting this product after myocarditis has improved to Grade 0-1 after treatment is unknown.

4.4.3 Famitinib Dose Adjustment

Famitinib dose adjustments include: dose interruption, dose reduction (20 mg down to 15 mg), and dose discontinuation. Once the famitinib administration method is adjusted during the study, it will not be allowed to be reversed. The maximum allowed interruption period for famitinib is 4 weeks (calculated from the actual time of the last dose). Famitinib dose adjustment guidelines are shown in the table below.

**Adverse Events**	**Grading**	**Whether The Dose Is Interrupted**	**Dose Adjustment Rules**
Hematology	Grade 1, 2	No	—
	Grade 3	Yes	• For the first occurrence, after the adverse reaction recovers to ≤ Grade 2, then the treatment should be continued at the original dose.<br>• For the second occurrence, after the adverse reaction recovers to ≤ Grade 2, the dose should be reduced to 15 mg.<br>• If it recurs after dose reduction, the investigator should assess the risk/benefit of the subject continuing to use famitinib and determine whether the treatment should be permanently discontinued.
	Grade 4	Yes	• After the adverse reaction recovers to ≤ Grade 2, the dose should be reduced to 15 mg.<br>• If Grade 4 toxicity recurs, the treatment should be permanently discontinued.
Non-hematological	Grade 1	No	—
	Grade 2 (lasting ≥7d)	Yes	• Once adverse reactions recover to ≤ Grade 1, the treatment should be continued at the original dose.
	Grade 3	Yes	• For the first occurrence, after the adverse reaction recovers to ≤ Grade 1, then the treatment should be continued at the original dose.<br>• For the second occurrence, after the adverse reaction recovers to ≤ Grade 1, the treatment should be reduced to 15 mg.<br>• •If it recurs after dose reduction, the investigator

			should assess the risk/benefit of the subject continuing to use famitinib and determine whether • the treatment should be permanently discontinued.
	Grade 4	Yes	• Once adverse reactions recover to ≤ Grade 1, the dose should be reduced to 15 mg.<br>• If Grade 4 toxicity recurs after dose reduction, the treatment should be permanently discontinued.

The following describes the measures to be taken for famitinib in the following special circumstances. If necessary, the investigator should consult with the relevant departments for specialist consultation:

Hemorrhage

- For Grade 3 or 4 bleeding events, appropriate treatment should be administered, and Famitinib treatment should be permanently discontinued.

Thrombosis/Embolism

- For arterial thrombotic events of any grade, Famitinib treatment should be permanently discontinued.
- For Grade 4 venous thrombosis, famitinib treatment should be permanently discontinued.
- For Grade 3 venous thrombosis, famitinib treatment should be interrupted. If the planned therapeutic dose of anticoagulant therapy is ≤2 weeks, Famitinib treatment should be interrupted until the end of anticoagulant therapy. If the planned therapeutic dose of anticoagulant therapy is >2 weeks, famitinib treatment should be interrupted for 2 weeks, and the study drug treatment can be restarted during anticoagulant therapy only after the following criteria are met:
  - The INR value should be within the target range (usually between 2 and 3) before restarting study drug treatment.
  - The subject must not have had any Grade 3 or 4 bleeding events since entering the study.
  - The subject must not have shown evidence of tumor invasion or proximity to large blood vessels in previous tumor assessments.

Note: Therapeutic dose anticoagulant therapy is defined as a gradual increase in the dose of warfarin or other anticoagulants to a level that maintains an INR of at least 1.5 (usually between 2 and 3). The dose of warfarin should be recorded in the eCRF, and subjects receiving anticoagulant therapy should have their INR monitored throughout the treatment period.

Hypertension:

Subjects should have their blood pressure measured regularly to monitor the onset and worsening of hypertension.

- Grade 1: Prehypertension (systolic blood pressure 120-139 mmHg, diastolic blood pressure

80-89 mmHg), no intervention required.

- Grade 2: Stage 1 hypertension (systolic blood pressure 140-159 mmHg, diastolic blood pressure 90-99 mmHg), medical intervention required. Repeated or persistent (≥ 24 hours), symptomatic increase in systolic blood pressure >20 mmHg or previous increase from normal range to ≥140/90 mmHg which can be treated with one antihypertensive medication.
- Grade 3: Stage 2 hypertension (systolic blood pressure ≥ 160 mmHg, diastolic blood pressure ≥100 mmHg), which requires more than one antihypertensive medication or higher intensity treatment than previously. For persistent or symptomatic hypertension, famitinib treatment should be paused; if hypertension remains uncontrolled after 4 weeks of treatment, famitinib treatment should be permanently discontinued.
- Grade 4: Life-threatening (malignant hypertension or persistent neurological damage, hypertensive crisis) acute intervention, life-threatening (e.g., hypertensive crisis). If Grade 4 hypertension occurs, famitinib treatment should be permanently discontinued. The dosage of antihypertensive medication used should be recorded at each visit.

Proteinuria:

Routine urinalysis should be performed according to the study flowchart. If:

- < 2+, the famitinib administration should be continued as planned, no further examination is required.
- ≥ 2+, the 24-hour urine protein should be immediately monitored. 24-hour urine protein:
  - < 1g: The famitinib administration should be continued as planned
  - 1-3.4g: The famitinib administration should be interrupted until 24-hour urine protein is < 1g, then the famitinib administration is continued.
  - ≥3.5g or nephrotic syndrome develops, the famitinib administration should be permanently discontinued.

4.5 Concomitant Treatment

4.5.1 Hematologic Support

Colony-stimulating factors (G-CSF or GM-CSF) or erythropoietin can be used prophylactically during treatment to prevent AEs such as neutropenia.

4.5.2 Treatment of Non-Hematologic Toxicities

Supportive therapies such as antiemetics, antibiotics, analgesics, antihypertensive drugs, and blood products can be used. Mouthwash can be used to treat or prevent stomatitis.

Unconventional therapies (e.g., herbal remedies or acupuncture) and vitamins/minerals may be used if the investigator believes they will not affect the study endpoints. Patients may receive bisphosphonate therapy for bone metastases during treatment. Palliative radiotherapy to a small

area (radiation area must be <5% of the bone marrow area) is permitted for bone metastases with pain that cannot be effectively controlled by systemic therapy or local analgesia. Clinical comorbidities and all types of AEs that occur should be actively treated and managed. All concomitant medications used should be recorded in the CRF.

4.5.3 Prohibited Medications During the Study

Subjects are prohibited from receiving the following treatments during the study period:

- Systemic chemotherapy and biotherapy (including anti-tumor drugs with immunomodulatory effects, including but not limited to interferon, interleukin-2, thymosin, immune cell therapy, etc.);
- Immunotherapy not specified in this protocol;
- Study drugs other than those specified in this study, including modern traditional Chinese medicine preparations approved by the NMPA for the treatment of lung cancer (including but not limited to Xihuang pills, Jinlong capsules, Kanglaite injection, Aidi injection, Kang'ai injection, and Xiao'aiping tablets, etc.);
- Live vaccines within 28 days before the first dose of the study drug and during the study, including but not limited to: measles, mumps, rubella, varicella, yellow fever, rabies, BCG, and typhoid (oral) vaccines. Inactivated viral vaccines administered by injection for seasonal influenza are permitted; however, live attenuated influenza vaccines administered intranasally (e.g., FluMist®) are not permitted;
- Corticosteroids; inhaled steroids used as part of maintenance therapy for asthma or chronic obstructive pulmonary disease (COPD) are permitted. Corticosteroids for the management of potential irAEs are permitted. Physiologic doses of corticosteroids are permitted. Prophylactic corticosteroids to avoid allergic reactions (e.g., contrast media) are permitted.
- Subjects who, based on the investigator's assessment, require clinical management with any of the above treatments should be excluded from the study. Subjects may receive other drug treatments deemed medically necessary by the investigator. It is very important for the investigator to review each medication (prescription and over-the-counter) that the subject is taking prior to study entry and at each study visit.
- At each visit, the subject must be questioned about any new medications taken since the last visit;
- To reduce the risk of drug interactions, every effort must be made to limit the number of concomitant medications used to those truly necessary;
- During the administration period, medications with hepatotoxicity (i.e., medications with warnings of hepatotoxicity in the product instructions) should be avoided. Investigators are

encouraged to review the potential hepatotoxicity of each medication by searching the website www.livertox.nih.gov.

- *In vitro* liver microsomal metabolism studies have shown that the metabolism of famitinib has certain species differences. The N-deethylation and N-dideethylation metabolism of famitinib are mainly catalyzed by CYP3A4, CYP3A5, and CYP1B1; monohydroxylation metabolism is mainly catalyzed by CYP1A2 and CYP1B1; and N-oxidation metabolism is catalyzed by FMO1. *In vivo* metabolism studies in rats showed that the metabolic pathways of famitinib are mainly N-deethylation, N-dideethylation, hydroxylation, N-oxidation, and hydroxylated glucuronide conjugation.
- Given that famitinib is mainly metabolized by the liver enzyme CYP3A4, subjects using famitinib should use CYP3A4 inducers (dexamethasone, carbamazepine, rifampicin, and phenobarbital) and inhibitors (ketoconazole, itraconazole, erythromycin, and clarithromycin) with caution during treatment. They should also use CYP3A4 substrates (simvastatin, ciclosporin, and pimozide) with caution, as well as other drugs metabolized by CYP3A4 (such as benzodiazepines, dihydropyridines, calcium channel blockers, and HMG-CoA reductase inhibitors). Caution should also be exercised with CYP2C9 substrates (diclofenac, phenytoin, piroxicam) and CYP2C19 substrates (diazepam, imipramine, lansoprazole).
- Given that a 50 mg/kg dose of famitinib can cause QT interval prolongation toxicity in anesthetized cynomolgus monkeys, subjects using famitinib should use drugs that prolong the QT interval with caution during treatment. These mainly include, but are not limited to, the following categories of drugs: antibacterial drugs (clarithromycin, azithromycin, erythromycin, roxithromycin, metronidazole, moxifloxacin); antiarrhythmic drugs (quinidine, sotalol, amiodarone, disopyramide, procainamide); antipsychotic drugs (risperidone, fluphenazine, droperidolum, haloperidol, thioridazine, pimozide, olanzapine, clozapine); antifungal drugs (fluconazole, ketoconazole); antimalarial drugs (mefloquine, chloroquine); antidepressant drugs (amitriptyline, imipramine, clomipramine, dothiepin, doxepin).

4.6 Study Procedure

Each subject will complete this clinical study after signing the informed consent form, going through the screening period, treatment period, and end of treatment stages. Before starting the study, the subject must read and sign the informed consent form approved by the Ethics Committee (EC). All study procedures must be performed within the time window specified in the study flow chart. All indicators and examination times are not affected by the length of

treatment discontinuation and are carried out in each treatment cycle as prescribed.

4.6.1 Screening Period

Subjects must sign the informed consent form before undergoing any study-specified screening procedures.

Unless otherwise specified, the following screening procedures must be completed within 28 days prior to the start of study drug treatment:

The screening visit procedures are shown in Table 5.

Table 5 Screening Visit Procedures

	Screening Period		Remarks
	-28-1 days	-7-1 days	
ICF	X		Subjects who do not meet the relevant criteria of this study (screening failure) may be re-screened.
Inclusion/Exclusion Criteria	X		
Demographics/Smoking History/Past Medical History/Prior Medication	X		Prior medication history includes treatment for the initial diagnosis, including chemotherapy, radiotherapy, and surgery. The date of the last anti-tumor treatment must be recorded.
Sample Collection, Pathological Diagnosis, and Genetic Testing	X		
Vital Signs	X		Vital signs include: body temperature, pulse, respiratory rate, and blood pressure. Refer to Section 6.1.4
Weight, Height	X		
Physical Examination	X		Refer to Section 6.1.2
Concomitant Diseases	X		
ECOG Performance Status	X		
12-Lead ECG		X	Refer to Section 6.1.4
Complete Blood Count /Blood Biochemistry/Urinalysis/Stool Examination		X	Refer to Section 6.1.1
Coagulation Function		X	
Pregnancy Test		X	Serum pregnancy test
Echocardiogram		X	Refer to Section 6.1.7
Cardiac Enzyme Panel		X	Refer to Section 6.1.7
Thyroid Function		X	Refer to Section 6.1.7
Hypothalamic-Pituitary-Adrenal Axis Evaluation	X		Refer to Section 6.1.7
HIV, HBV, and HCV	X		Refer to Section 6.1.7

Pulmonary Function Test	X		Refer to Section 6.1.7
AE Assessment	X	X	All AEs/SAEs from the time of signing the ICF until the end of the safety follow-up period should be recorded in the CRF.
Concomitant Medications	X	X	All concomitant medications used within 28 days prior to the first dose of study drug administration until 90 days after the end of treatment should be recorded in the CRF.
Tumor Imaging Assessment	X		Refer to Sections 5.3 and 5.4
Quality of Life Score		X	

4.6.2 Treatment Period

The treatment period visit procedures are shown in Table 6.

Table 6 Treatment Period Visit Flow Chart

	Treatment Period		Remarks
	Cycle 1 D1±3	Subsequent Cycle D1±3	
Vital Signs	X	X	Vital signs include: body temperature, pulse, respiratory rate, and blood pressure. Refer to Section 6.1.5
Weight, Height	X	X	
Physical Examination	X	X	Refer to Section 6.1.2
ECOG Performance Status	X	X	
12-lead ECG	X	X	Refer to Section 6.1.3
Complete Blood Count /Blood Biochemistry/Urinalysis/Stool Examination		X	Refer to Section 6.1.1
Coagulation Function		X	
Pregnancy test			Serum pregnancy test
Echocardiogram			Refer to Section 6.1.7
Cardiac enzyme panel		Additional tests when clinically indicated	Refer to Section 6.1.7
Thyroid Function		Once every cycle for cycles 2-4, and then once every 2 cycles thereafter.	Refer to Section 6.1.7
Hypothalamic-Pituitary-Adrenal Axis Evaluation		Once every cycle for cycles 2-4, and then once every 2 cycles thereafter.	Refer to Section 6.1.7
Pulmonary Function Test		Additional tests when clinically indicated.	Refer to Section 6.1.7

AE Assessment	X	X	All AEs/SAEs from the time of signing the ICF until the end of the safety follow-up period should be recorded in the CRF.
Combination Therapy	X	X	All combination therapies used within 28 days prior to the first dose of study drug administration until 90 days after the end of treatment should be recorded in the CRF.
Tumor Imaging Assessment	Every 6 weeks (±7 days) within 12 months after the first dose, and every 12 weeks (±7 days) after 12 months following the first dose.		Refer to Sections 5.3 and 5.4
Quality of Life Score		Performed at each imaging assessment	

4.6.3 End of Treatment

End of treatment is defined as confirmation of disease progression or withdrawal from the study.

Table 7 End of Treatment Visit Flow Chart

	End of Treatment ± 3 days	Remarks
Vital Signs	X	Vital signs include: body temperature, pulse, respiratory rate, and blood pressure. Refer to Section 6.1.4
Weight, Height	X	
Physical Examination	X	Refer to Section 6.1.2
ECOG Performance Status	X	
12-Lead ECG	X	Refer to Section 6.1.4
Complete Blood Count /Blood Biochemistry/Urinalysis/Stool Examination	X	Refer to Section 6.1.1
Coagulation Function	X	
Pregnancy Test	X	Serum pregnancy test
Echocardiogram	X	Refer to Section 6.1.7
Cardiac Enzyme Panel	X	Refer to Section 6.1.7
Thyroid Function	X	Refer to Section 6.1.7
Hypothalamic-Pituitary-Adrenal Axis Evaluation	X	Additional tests when clinically indicated.
AE Assessment	X	All AEs/SAEs from the time of signing the ICF until the end of the safety follow-up period should be recorded in the CRF.
Concomitant Medications	X	All concomitant medications used within 28 days prior to the first dose of study drug administration until 90 days after the end of

		treatment should be recorded in the CRF.
Tumor Imaging Assessment	X	Refer to Sections 5.3 and 5.4
Quality of Life Score	X	

4.6.4 Safety Follow-Up

Safety follow-up will be conducted within 90±7 days after discontinuation (refer to Table 8).

Table 8 Safety Follow-up Procedures

	Safety Follow-Up		Remarks
	30±7 days	30n±7 days (n = 2, 3)	
Vital Signs	X		Vital signs include: body temperature, pulse, respiratory rate, and blood pressure. Refer to Section 6.1.5
Body Weight	X		
Physical Examination	X		Refer to Section 6.1.2
Symptoms of Intracranial Hypertension	X		Refer to Section 6.1.3
ECOG Performance Status	X		
12-Lead ECG	X		
Complete Blood Count /Blood Biochemistry/Urinalysis/Stool Examination	X		Refer to Section 6.1.1
Coagulation Function	X		
Thyroid Function	X		
AE Assessment	X	X	All AEs/SAEs from the time of signing the ICF until the end of the safety follow-up period should be recorded in the CRF.
Concomitant Medications	X	X	All concomitant medications used within 28 days prior to the first dose of study drug administration until 90 days after the end of treatment should be recorded in the CRF.
Vital Status	X	X	
Subsequent Anti-Tumor Therapy	X	X	
Imaging Assessment	X		For patients who discontinue treatment for reasons other than progressive disease (PD) based on imaging, imaging evaluations should be conducted according to the scheduled imaging assessment time points until one of the following events occurs: initiation of new anti-tumor therapy, PD, withdrawal of informed consent (ICF) by the subject, or death.

4.6.5 Survival Follow-up

Survival follow-up will be conducted every 3 months after the safety follow-up is completed, and telephone follow-up is acceptable.

4.7 Drug Management

4.7.1 Drug Management

Camrelizumab: Camrelizumab should be refrigerated at 2-8℃, protected from light and kept dry, and should not be frozen. All trial drugs are transported to each study site via cold chain and should be kept and dispensed by designated personnel.

The investigational drug should be stored in a refrigerator that can only be opened by authorized personnel. After receiving the drug, the investigator should confirm that the drug transport temperature is within the specified range, check and sign for it, and store it at the specified temperature. If temperature abnormalities occur during transportation, during storage at the study site, or at any other time, the drug should be immediately transferred to the specified temperature for mitigation, temporarily withheld from subjects, and promptly reported to the principal investigator for further instructions.

The study drug camrelizumab can only be used for this trial and must not be used for purposes other than those specified in this protocol. The investigator must commit to not providing the investigational drug to any personnel unrelated to this trial.

Camrelizumab that has been discontinued for special reasons should be stored under the same storage conditions until it has been checked and verified by the monitor, and then arrangements should be made for its return.

Famitinib: Famitinib should be protected from light, sealed, and stored below 25℃. According to GCP requirements, the study drug (famitinib) will be uniformly stored, dispensed, and retrieved by the trial site. From the first day of the first cycle of receiving the donated drug, the research personnel should, on the first day of each subsequent cycle:

- Specify the number of famitinib capsules to be dispensed to the patient for the next treatment cycle,
- Record this information on the study drug CRF page and in the drug dispensing log.
- Instruct patients to comply with the treatment plan and return all unused capsules and all drug packaging boxes, including empty boxes and capsule boxes, at each visit.

4.7.2 Drug Retrieval and Destruction

In this study, used and partially used drug containers, vials, infusion bags, and syringes can be destroyed on-site according to the applicable guidelines and operating procedures established

by the study site and local institutions.

Unused drugs should be retrieved or destroyed according to the applicable guidelines and operating procedures established by the study site and local institutions, unless the drug contents have significant safety issues that require immediate destruction according to local regulations.

4.7.3 Study Drug Records

Designated personnel at the study site are required to keep timely records of the receipt, distribution, use, inventory, destruction, retrieval, and damage of study drugs in accordance with relevant regulations and guidelines.

5. Clinical Evaluation

5.1 Primary Efficacy Endpoint

- Objective Response Rate (ORR): defined as the proportion of subjects whose confirmed best overall response (BOR) is complete response (CR) or partial response (PR), evaluated by the investigator according to RECIST version 1.1. Subjects must have measurable tumor lesions at baseline. A first finding of CR/PR should be confirmed by a subsequent tumor assessment at least 4 weeks later.

5.2 Secondary Efficacy Endpoints

1) Disease Control Rate (DCR): defined as the proportion of subjects whose confirmed best overall response (BOR) is complete response (CR), partial response (PR) or stable disease (SD), evaluated by the investigator according to RECIST version 1.1.
2) Duration of Response (DoR): defined as the time from the date of the first documentation of confirmed CR or PR to the date of disease progression or death due to any cause, whichever occurs earlier.;
3) Progression-Free Survival (PFS): The time from treatment initiation to disease progression or death due to any cause, whichever occurs earlier.
4) Overall Survival (OS): The time from t treatment initiation to death from any cause. Patients still alive at the time of analysis will be censored at their last known survival date.
5) Subject Quality of Life Score: Assessed and recorded according to the Quality of Life Questionnaire (EOETC QLQ-C30).
6) Adverse Events: Record all adverse events from the signing of the ICF until the end of the safety follow-up period, and determine whether they are related to the study drug.

5.3 Imaging Scan Locations

The imaging methods used for tumor assessment at baseline must be consistent with those used for each subsequent follow-up assessment.

Computed tomography (CT) or magnetic resonance imaging (MRI) scans are recommended.

Based on the presenting signs and symptoms of each subject, examinations of other involved sites will be performed. Baseline assessments must include chest, upper abdomen (liver, gallbladder, pancreas, and spleen), brain, and all known or suspected sites of disease. Subsequent clinical tumor imaging evaluations should include the chest and upper abdomen. The investigator may increase the frequency of imaging monitoring based on the actual situation.

5.4 Assessment Time Points

Baseline tumor assessment will be performed within 28 days prior to the first dose. Imaging data obtained before signing the informed consent can be used for screening tumor assessment as long as it meets the protocol requirements. Imaging assessments will be performed every 6 weeks (±7 days) after the first dose during the study until imaging-based PD is documented. For subjects with the first documented CR or PR and the first documented imaging-based PD, an additional imaging assessment should be performed 4 weeks (±7 days) later to confirm the response or PD.

Criteria for Subject's Subsequent PD:

- The sum of the diameters of target lesions increases by ≥20% compared to the nadir (the smallest sum of diameters, which may occur at baseline or subsequent visits) and the absolute increase is at least 5mm on two consecutive visits, and/or
- At the confirmatory PD time point, non-target lesions or new lesions have significantly progressed (worsened to the extent that even if the target lesions show CR, PR, or SD, the overall tumor burden has increased enough to warrant discontinuation of treatment) compared to the first time point when non-target lesion progression or new lesions were found, and/or
- Additional new lesions appear at the confirmatory PD time point compared to the first time point when new lesions are found.
- If PD is not confirmed, the study drug treatment is continued and imaging assessments are performed until subsequent PD.
- Subjects with confirmed PD must discontinue treatment. For patients who discontinue treatment for reasons other than PD based on imaging, imaging evaluations should be conducted according to the scheduled imaging assessment time points until one of the following events occurs: initiation of new anti-tumor therapy, PD, withdrawal of ICF by the subject, or death.
- Subjects judged to be clinically unstable should discontinue study treatment after the investigator's initial assessment of imaging-based PD is confirmed at the center, and

confirmation of PD by repeat imaging is not required.

The definition of clinically stable is as follows:

- No clinically significant signs or symptoms suggestive of disease progression (including worsening laboratory values) have occurred.
- ECOG Performance Status does not decrease
- No rapid disease progression
- No progressive tumor growth requiring other urgent medical intervention (e.g., spinal cord compression) has occurred at critical anatomical sites.

If the investigator is unable to determine whether the disease has progressed, especially for non-target lesions and new lesions, the subject may continue treatment and the disease status should be reassessed when clinical signs appear or at the next scheduled assessment time point. If the repeat scan confirms PD, the date of progression should be the date of the initial finding.

Interruption of one or more study drugs does not affect the frequency of RECIST 1.1 assessment.

6. Safety Assessment

6.1 Safety Indicators

6.1.1 Routine Laboratory Safety Assessment Indicators

Table 9. Routine Laboratory Safety Assessment

Complete Blood Count	RBC, HGB, HCT, WBC, PLT, LYM, ANC, MONO, EOS, and BASO
Coagulation Function	TT, PT, APTT, and INR
Blood Biochemistry	TBIL[a]、ALT、AST、γ-GT 、ALP、ALB、TP、LDH、BUN、UREA、Cr、Na、K、Cl、Mg、Ca、P
Urinalysis	PH, UALB, UPRO, URBC, and UGLU
Stool Examination	Occult blood

a. If TBIL ≥ 2×ULN (and there is no evidence of Gilbert's syndrome), then direct bilirubin and indirect bilirubin should be measured separately.

6.1.2 Physical Examination

A complete physical examination includes: general condition, respiratory, cardiovascular, abdomen, skin, head and neck (including ears, eyes, nose, and throat), lymph nodes, thyroid, musculoskeletal (including spine and extremities), genital/rectal, and neurological system assessment.

6.1.3 12-Lead ECG

A 12-lead resting ECG will be analyzed at a local laboratory according to the visit schedule.

A 12-lead ECG should be performed on each subject after at least 5 minutes of rest in a supine position. All 12-lead ECGs should be recorded while the subject is resting in a supine position. Further ECG examinations will be performed when clinically indicated, such as when cardiac-

related AEs occur. The investigator will complete the ECG assessment on the day of the examination and record the assessment results on the electrocardiogram. The same assessment method should be used throughout the study.

The ECG will be recorded at a speed of 25 mm/sec. The investigator should assess all ECGs according to the categories of clinically significant abnormalities/no clinically significant abnormalities. If there are clinically significant abnormal results, the investigator should record the results as AEs in the CRF.

6.1.4 Vital Signs

Vital signs will be checked as described in the study visit schedule. Vital signs include body temperature, pulse rate, respiratory rate, and blood pressure; investigators may perform additional vital sign assessment and monitoring at their discretion based on standard clinical practice or clinical need.

Pulse, blood pressure, body temperature, and respiration will be collected during the screening period, before the planned infusion of camrelizumab on the day of administration, at the end of treatment, and during safety follow-up.

Blood pressure monitoring: Smoking and coffee are prohibited within 30 minutes before each blood pressure measurement. Rest quietly for at least 10 minutes. Measurements should be taken in a sitting position with the elbow at the same level as the heart. Each blood pressure measurement should be taken on the same side.

6.1.5 Height and Weight

Height measurement is only performed during the screening period. Weight measurement is required during the screening period, before each visit and administration of camrelizumab, at the end of treatment, and during safety follow-up.

6.1.6 Pregnancy Test

Female subjects of childbearing potential will undergo a serum human chorionic gonadotropin (HCG) pregnancy test within seven days prior to the first dose of study drug. If the result is positive, the subject is ineligible/must discontinue participation in the study. If pregnancy is suspected during the study, a re-examination should be performed.

6.1.7 Other Safety Checks

1) Hepatitis B Panel: HBsAg, HBsAb, HBcAb, HBeAg, HBeAb.
2) HIV antibody, HCV antibody
3) Thyroid function: T3, T4, TSH, FT3 and FT4
4) Myocardial enzyme spectrum: LCK, CK-MB
5) Hypothalamic-Pituitary-Adrenal Axis Examination: including CRH, plasma ACTH and

adrenocortical hormone determination. Adrenocortical hormone determination includes serum cortisol, UFC, 17-KS and 17-KGS (only some of these indicators may be checked, required during the screening phase, and additional checks are performed when clinically indicated)

6) Echocardiogram
7) Pulmonary function test: FVC, FEV1, FEV1/FVC

6.2 Safety Parameters

Safety parameters include adverse events related to study treatment; serious adverse events; abnormal laboratory test values; unexpected safety events, clinical manifestations, severity, time of occurrence, duration, treatment methods and prognosis of adverse events of special interest;

6.3 Abnormal Laboratory Tests

Laboratory test blood/urine/other samples will be collected according to the study flow chart and tested in the central laboratory.

Abnormal results of the following laboratory tests should be recorded on the corresponding CRF non-serious AE page or SAE report form (written or electronic report form):

- All laboratory test results that are clinically significant or meet the SAE definition
- All abnormal laboratory test results that require the subject to discontinue or interrupt the study drug
- All abnormal laboratory test results that require the subject to use specific symptomatic treatment.

The reporting investigator should preferably use clinical terms rather than laboratory test terms (e.g., anemia, rather than low hemoglobin).

6.4 Safety Assessment Criteria

Assessment of AEs includes type, incidence, severity (graded according to NCI CTCAE 5.0), time of onset and end, relationship to study treatment, and outcome.

7. Adverse Events

7.1 Adverse Event (AE)

AE definition: An AE is any untoward medical occurrence in a clinical trial subject administered a pharmaceutical product and which does not necessarily have a causal relationship with this treatment.

An AE can be any unfavorable and unintended sign, symptom, or disease temporally associated with the use of a medicinal (investigational) product, whether or not considered related to the medicinal (investigational) product. At least the following situations are included:

1) Pre-existing medical conditions/diseases (present before entering the clinical trial) should only be recorded as AEs if they worsen (including worsening of symptoms, signs, or abnormal laboratory findings) after starting the trial drug;

2) Any new AE: Any new adverse medical condition (including symptoms, signs, or newly diagnosed diseases);

3) Clinically significant abnormal laboratory values or results.

Diagnostic or therapeutic invasive (e.g., surgery) or non-invasive procedures should not be reported as AEs. However, if the medical condition leading to the procedure meets the definition of an AE, it should be reported. For example, acute appendicitis occurring during the AE reporting period should be reported as an AE, while the appendectomy performed should be recorded as the treatment for the AE.

The investigator should document in detail any AEs experienced by the subjects, including: AE name, start and end dates, severity (graded according to NCI CTCAE version 5.0), relationship of the AE to the study drug, duration, measures taken for the trial drug due to the AE, outcome of the AE, and whether it is a SAE. This information should be accurately recorded in the CRF. All AEs/SAEs should be followed up until the end of the safety follow-up period or until they resolve, return to baseline or grade 0-1, reach a stable state, or have a reasonable explanation (e.g., loss to follow-up, death).

7.2 AE Severity Grading Criteria

AEs are graded from 0-5 according to the NCI Common Terminology Criteria for Adverse Events (NCI-CTCAE 5.0). If an AE is not listed in the NCI toxicity grading criteria, it can be assessed according to the following criteria:

Grade I: Mild; asymptomatic or mild clinical symptoms; clinical or laboratory findings only; no treatment required;

Grade II: Moderate; requires minor, local, or non-invasive treatment; limitation of age-appropriate instrumental Activities of Daily Living (ADL), instrumental ADL refers to cooking, shopping, making phone calls, counting money, etc.;

Grade III: Severe or medically significant but not immediately life-threatening; hospitalization or prolongation of hospitalization; resulting in disability; limitation of Self-care ADL. Self-care ADL refers to: bathing, dressing, undressing, eating, toileting, taking medication, etc., not bedridden;

Grade IV: Life-threatening; urgent intervention indicated

Grade V: Death related to AEs.

The distinction between the severity and seriousness of an adverse event should be noted. For

example, a “severe headache” may be severe in intensity but would not be classified as an SAE unless it meets the criteria for an SAE.

7.3 Serious Adverse Events (SAE)

1) An SAE is defined as a medical event occurring during a clinical trial that requires hospitalization or prolongation of existing hospitalization, results in disability, affects work ability, is life-threatening or results in death, or leads to congenital anomalies.

Adverse events that meet one or more of the following criteria are considered SAEs:

- Events leading to death;
- Life-threatening (the term “life-threatening” refers to a situation where the subject is at immediate risk of death at the time the event/reaction occurs; it does not mean that death may occur only if the event/reaction further deteriorates);
- Requires hospitalization or extension of existing hospital stay;
- Events leading to permanent or severe disability/dysfunction/impairment of work ability;
- Congenital anomalies or birth defects;
- Other important medical events (referring to those events/reactions that, while not immediately life-threatening, fatal, or requiring hospitalization, may pose a hazard to the subject based on reasonable medical and scientific judgment, or may require intervention [such as medication or surgery] to prevent the serious consequences listed in the above definition).

2) Hospitalization

Hospitalization (even if less than 24 hours) during a clinical trial should be considered an SAE, except for hospitalization or prolonged hospitalization due to the following circumstances, which do not need to be reported as SAEs:

- Rehabilitation institutions
- Nursing home
- Routine emergency room admission
- Same-day surgery (e.g., outpatient/same-day/non-inpatient surgery)
- Social reasons (medical insurance reimbursement, etc.)
- Hospitalization or prolonged hospitalization unrelated to AEs is not an SAE. This may include, but is not limited to, the following situations:
  - Hospitalization due to a pre-existing condition without the occurrence of new AEs or worsening of the pre-existing condition (e.g., hospitalization for examination of persistent laboratory abnormalities present before the trial);
  - Hospitalization for administrative reasons (e.g., annual routine physical examination);

- Hospitalization stipulated in the clinical trial protocol (e.g., operations performed as required by the protocol);
- Elective hospitalization unrelated to AEs (e.g., elective surgery);
- Pre-scheduled treatments or surgical procedures should be documented in the overall trial protocol and/or the individual subject's baseline data;
- Hospitalization solely due to the use of blood products.

3） Undergoing other anti-tumor treatments:

If the subject starts other anti-tumor treatments before the end of the safety follow-up period, the reporting period for non-fatal AEs suspected to be unrelated to the trial drug ends at the start of the new anti-tumor treatment. If death occurs during the safety follow-up period, it must be reported as an SAE regardless of whether the subject received other treatments.

4） Drug overdose:

Drug overdose refers to the subject taking an additional dose of the study drug within 24 hours (the specific time is adjusted according to the specific protocol), which is higher than the dose prescribed by the investigator. Drug overdose combined with AE/SAE needs to be reported to the trial drug manufacturer as a serious adverse event.

7.4 Definition of Unexpected Safety Events

Expected AEs are common AEs collected from completed/ongoing studies, and such events should be collected in a systematical (using a grading system) and standardized way. The method for determining expected AEs should be described. Expectancy indicates that such AEs have been observed in previous studies, not predicted based on the chemical properties of the drug itself.

Unexpected safety events are events or outcomes that meet the following criteria:

The Camrelizumab Investigator's Brochure and the post-marketing instructions for pemetrexed and carboplatin will be used as reference materials for the expectedness assessment of adverse events. If the nature, severity, specificity, and outcome of an event are inconsistent with the descriptions in the Investigator's Brochure and/or post-marketing instructions, the event is considered unexpected.

7.5 Collection and Follow-up of AEs/SAEs

AE information should be collected from the time the subject signs the informed consent form until the end of the safety follow-up period, which is 90 days after the last dose of camrelizumab or the start of new anti-tumor therapy (whichever comes first).

If death occurs during the safety follow-up period, it must be reported as an SAE. After the safety follow-up period, the investigator only needs to report SAEs related to the study drug to the drug safety department of the trial drug manufacturer.

All AEs/SAEs should be followed up until the end of the safety follow-up period or until they disappear, resolve to baseline levels or ≤ Grade 1, reach a stable state, or have a reasonable explanation (e.g., loss to follow-up, death), and be recorded accurately and promptly in the CRF. The investigator should inquire about AEs/SAEs that have occurred since the last visit at each visit and provide follow-up information promptly based on the query requests received.

7.6 Assessment of the Relationship Between AEs and the Study Drug

The investigator should comprehensively assess the relationship between the AE and the study drug, such as whether the occurrence of the AE has a reasonable temporal relationship with the drug administration, the characteristics of the study drug, the toxicological and pharmacological effects of the study drug, whether the subject is using other concomitant medications, the subject's underlying disease, medical history, family history, and deactivated and reactivated reactions, etc. And the possible relationship between AEs and the study drug should be assessed according to the five-level classification of "definitely related, possibly related, possibly unrelated, and unable to evaluate".

The judgment criteria can refer to the following table:

Correlation Evaluation	Temporal relationship	Whether It Is Known	Deactivation	Reactivation	Other Explanations
Definitely related	+	+	+	+	—
Possibly related	+	±	±?	?	±?
Possibly unrelated	—	—	±?	?	±?
Definitely unrelated	—	—	—	—	+
Unable to evaluate	Essential data for evaluation could not be obtained				

1. + indicates positive; - indicates negative; ± indicates difficult to judge; ? indicates unknown.
2. Temporal relationship: whether there is a reasonable temporal relationship between drug administration and the occurrence of adverse reactions.
3. Whether it is known: whether the adverse reaction is consistent with the known adverse reaction type of the drug.
4. Deactivation: whether the adverse reaction disappears or is reduced after drug discontinuation or dose reduction.
5. Reactivation: whether the same adverse reaction reappears after re-administration of the suspected drug.

6. Other explanations: whether the adverse reaction can be explained by the effects of concomitant medications, the progression of the subject's disease, the effects of other treatments, etc.

7.7 SAE Reporting Procedures

SAEs should be reported from the time the subject signs the informed consent form until the end of the safety follow-up period. If an SAE occurs after the start of the study drug, it should also be reported to the Hengrui drug safety department.

If an SAE occurs, whether it is the initial report or a follow-up report, the investigator must immediately complete the "Serious Adverse Event Report Form", sign and date it, and report it to the relevant departments and the drug safety department of the trial drug manufacturer (hengrui_drug_safety@hengrui.com) within 24 hours of the investigator becoming aware of it. All SAEs should be documented in detail, including symptoms, severity (refer to NCI-CTC 5.0 grading), relationship to each study drug, time of onset, time of treatment, measures taken for each study drug due to the SAE, follow-up time and method, and outcome. If the investigator believes that an SAE is not related to the study drug but is potentially related to the study conditions (e.g., discontinuation of prior therapy or comorbidities during the trial), this relationship should be explained in detail in the narrative section of the SAE report form. If the intensity of an ongoing SAE or its relationship to the study drug changes, a follow-up report should be submitted immediately. If the investigator believes that a previously reported SAE contains misinformation, a correction, withdrawal, or downgrade explanation can be made in the follow-up report and reported according to the SAE reporting procedures.

7.8 Unexpected Adverse Event Reporting

Unexpected AEs should be recorded in the Unexpected Adverse Event Form. The investigator is obligated to report unexpected AEs to the EC and the manufacturer of the study drug. The report should include the following information:

- Basic study information: study name, code, principal investigator's name, and ethics committee project number;
- Details or outcomes of the event;
- Justification for determining that the event is an unexpected AE;
- Description of protocol amendments or other corrective actions taken/recommended based on the unexpected AE.

To meet the requirements for timely reporting, unexpected AEs will be reported according to the following schedule:

- Unexpected AEs that are SAEs will be submitted to the ethics committee and the manufacturer of the study drug within 24 hours of the investigator becoming aware of them.
- Other unexpected adverse events, if the investigator believes they warrant special attention, should be submitted to the ethics committee and the manufacturer of the study drug within 24 hours of that determination.

7.9 Pregnancy Report

If a female subject becomes pregnant during the study, the subject will immediately discontinue the study drug and withdraw from the study. If the female partner of a male subject becomes pregnant during the study, the male subject may continue in the study.

The investigator should complete the "Clinical Trial Pregnancy Report/Follow-up Form" and report it to the relevant departments within 24 hours of becoming aware of the pregnancy. If the pregnancy occurs after the start of study drug administration and before the end of the safety follow-up period, the "Clinical Trial Pregnancy Report/Follow-up Form" should be completed and submitted to the drug safety department of the manufacturer of the study drug within 15 days of the investigator becoming aware of the pregnancy.

The investigator is required to follow up on pregnancy outcomes until 1 month after the mother gives birth, and report the results to the relevant departments and the drug safety department of the study drug manufacturer.

If the pregnancy outcome is stillbirth, spontaneous abortion, or fetal malformation, it will be considered an SAE and needs to be reported according to SAE reporting timelines.

If the subject experiences an SAE concurrently during pregnancy, a "Serious Adverse Event Report Form" must also be completed, and the SAE reporting procedures must be followed.

7.10 Disease Progression and Death Reporting

Disease progression is defined as worsening of the subject's condition caused by the indication under study. This includes progression based on imaging evidence, or progression of clinical symptoms and signs. New metastatic lesions of the primary tumor, or progression of existing metastatic lesions are both considered disease progression.

Life-threatening events caused by symptoms and signs of disease progression, requiring hospitalization or prolonged hospitalization, or resulting in permanent or severe disability/dysfunction/impaired work ability, congenital anomalies, or birth defects, are not reported as SAEs. If there is any uncertainty as to whether an SAE is caused by disease progression, it should be reported as an SAE.

In the study population of this trial, “disease progression” is an expected occurrence and should not be reported as an AE term. When disease progression occurs, the event used to confirm disease progression should be reported as an AE. For example, if a subject experiences epilepsy, which is determined to be related to brain metastasis, the AE term should be recorded as “epilepsy,” not “disease progression” or “brain metastasis.”

Death that is assessed by the investigator as possibly due to symptoms and signs of disease progression should be reported as an SAE.

However, the term “death” should not be used as an AE or SAE term, but rather as the outcome of the event. The event that caused or led to death should be recorded as the SAE. If the cause of death is unknown and cannot be determined at the time of reporting, the SAE term should be recorded as “death from unknown cause.”

8. Statistical Analyses

This section provides a technical elaboration for the analyses of the efficacy and safety data.

For continuous variables, descriptive statistics including number of subjects with non-missing values, mean, standard deviation, median, quartiles (Q1, Q3), minimum, and maximum will be presented. For categorical variables, frequencies and percentages will be presented. Time-to-events data will be analyzed by Kaplan-Meier method, and K-M plots will be presented if necessary. All the statistical analyses will be performed with SAS® Version 9.4 or above.

8.1 Analysis Population

This study will involve the following analysis sets:

1) Full Analysis Set (FAS):

All subjects who sign the informed consent and receive at least one dose of the study treatment constitute the full analysis set. The full analysis set is the primary analysis set for this study.

2) Per-Protocol Set (PPS):

The per-protocol set is a subset of the FAS, defined as subjects who have no major protocol deviations or deviations that significantly affect the study results during the study.

Major protocol deviations or deviations that are judged to have a significant impact on the study results generally include, but are not limited to:

- Violation of inclusion/exclusion criteria;
- Taking prohibited medications during the study;

The identification and determination of subjects with major protocol deviations or deviations that have a significant impact on the study results will be determined before database lock.

These subjects (if any) will be excluded from the PPS. Analysis based on the PPS will serve as secondary/supportive analysis for this study.

3) Safety Analysis Set (SS):

All subjects who receive at least one dose of study treatment constitute the safety set of this study. The safety analysis will be performed based on safety set.

4) Efficacy Analysis Set (EAS):

The Efficacy Analysis Set (EAS) will include all treated subjects who have baseline tumor assessment and at least one post-baseline tumor assessment according to RECIST version 1.1.

8.2 Statistical Methods

8.2.1 Subject Disposition

Subject disposition information, including but not limited to the number of subjects being enrolled, treated, withdrawing from the trial, and major protocol deviations occurring in patients, will be summarized. The reasons for screening failure and the reasons for withdrawal from the trial will also be summarized.

The number and percentage of subject in each analysis set (FAS, PPS, EAS, SS) will be summarized.

In addition, the duration of subject in study will also be summarized:

- Duration in study (months) = (Last day known to be alive – first treatment date + 1)/30.4375.

8.2.2 Demographic and Baseline Characteristics

Statistical description will be performed on demographic and baseline characteristics for FAS population. The demographic characteristics and disease diagnosis information as well as first-line chemotherapy information listed below will be summarized.

8.2.3 Analysis of Primary Efficacy Endpoint

FAS will be used as the primary analysis set for the primary efficacy analysis. The confirmed objective response rate (ORR) will be calculated and the corresponding 95% Clopper–Pearson confidence intervals (CI) will be presented. In addition, the confirmed best overall response (BOR) will be summarized.

8.2.4 Analysis of Secondary Efficacy Endpoints

The analysis of secondary efficacy endpoints in this study will be based on FAS, including DCR, DoR, PFS, and OS. For DCR, disease control rate will be calculated along with the corresponding 95% Clopper–Pearson CI. For time-to-event data of the secondary endpoints, Kaplan–Meier method will be adopted to estimate median time to event of PFS, DoR, TTR and OS, and the Brookmeyer–Crowley method will be used to estimate the corresponding 95% confidence intervals. The Kaplan-Meier curves will also be presented. The analysis will be performed mainly based on FAS.

Regarding the censoring rule of PFS in our study, if there is no baseline or post-baseline efficacy assessment, it will be censored at treatment initiation date; if subsequent anti-tumor therapy is initiated, PFS will be censored at the last adequate efficacy assessment date before the initiation of new anti-tumor therapy; if disease progression and death are not observed and without the initiation of new anti-tumor therapy, PFS will be censored at the last post-baseline adequate efficacy assessment date.

Descriptive statistics will be summarized for the disease-related symptom quality of life score (EOETC QLQ-C30) and its change from baseline to assess the change and relief of subjects' disease-related symptoms compared to baseline levels.

8.2.5 Safety Analyses

Safety analysis will be based on the safety set (actual treatment group).

Treatment-emergent adverse events (TEAEs) are defined as adverse events which occur or become more sever after receiving the first study treatment, whichever occurs later. If the CTCAE grade of an AE is missing, it will be considered as grade 3 AE. The safety analysis is limited to descriptive statistical summaries, including the number and percentage of subjects with:

- Any TEAEs
- TEAEs with CTCAE grade ≥ 3
- TEAEs leading to dose reduction/ drug interruption/drug delay/ drug discontinuation
- Serious TEAEs (TESAE)
- Any TRAEs (including TEAEs related to any study treatment)
- TRAEs leading to death
- TRAEs with CTCAE grade ≥ 3
- TRAEs leading to dose reduction/ drug interruption/drug delay/ drug discontinuation
- TRAEs leading to death
- Serious TRAEs (TRSAE)

- Immune-related TEAEs

The number and percentage of subjects reporting TEAEs will be reported, and they will be further be summarized by preferred term (PT) and severity (overall and for CTCAE grade ≥ 3). Moreover, TRAEs with an incidence of ≥10% will also be presented by PT and severity. A subject who reports multiple TEAEs with the same PT will only be counted once at the corresponding PT level with the most sever CTCAE grade. If necessary, the Kaplan–Meier method will be used to analyze the time to AE occurrence and duration of AE for AEs of special concern.

8.2.6 Compliance and Retention Analysis

This study defines poor compliance as situations where subjects, after enrollment, believe the treatment is ineffective and do not take the medication according to the prescribed dose and course, including:

Receiving unplanned doses and courses of study drug treatment;

Exceeding the stipulated delay in study drug treatment;

The proportion of subjects with poor compliance in the full analysis set will be calculated and summarized.

9. Ethical Considerations

9.1 Ethical Standards

The study will strictly adhere to the ethical principles for human medical research of the Declaration of Helsinki and relevant clinical trial regulations in China. The investigator is responsible for providing the clinical trial protocol, informed consent form, and information materials provided to the subjects to the EC to obtain independent approval documents for conducting this clinical study.

Approval documents from the EC must be obtained before the clinical study begins. The ethics committee's approval documents must be submitted to the investigator in writing, and the investigator will then provide a copy of the approval documents to the study sponsor. The ethics committee's approval documents must be accompanied by a list of all committee members who participate in the discussion of the approval documents and their respective responsibilities.

Without the agreement of both the study sponsor and the investigator, neither party may unilaterally modify this study protocol. Only to eliminate immediate hazards to subjects, may the investigator implement changes or deviate from the study protocol prior to obtaining approval from the EC. At the same time, the deviations or changes made, the reasons for them,

and the proposed protocol amendments should be submitted to the EC for review as soon as possible. The investigator must explain and document any deviations from the protocol.

During the clinical study, any issues related to the safety of the clinical study, such as changes to the clinical study protocol or the informed consent form, as well as SAEs in the clinical study, must be reported to the EC in a timely manner. The completion or early termination of the clinical study must also be reported to the EC.

All potentially serious breaches of regulations must be reported immediately. Serious breaches of regulations refer to violations of GCP conditions and principles related to the study or deviations from the study protocol that may have a significant impact on the safety or physical and mental health of the study subjects or the scientific value of the study.

Personnel involved in the implementation of this study must be appropriately qualified, and their educational background, training experience, and experience in performing their respective tasks must meet the requirements.

9.2 Informed Consent

9.2.1 Informed Consent Form and Other Written Information Required by Subjects

The informed consent form will describe in detail the study medication and the study process, and will fully explain the study risks to the subjects. Written informed consent must be obtained before administering the study medication.

All revisions to the informed consent form must also be approved by the EC, and the EC will decide whether subjects who have signed the previous version of the informed consent form need to sign the new version again.

The informed consent form should also state that records of the subject's personal identity must be kept confidential, but regulatory authorities may review the subject's information.

9.2.2 Informed Consent Process and Documentation

The informed consent process begins before the individual agrees to participate in the clinical study and continues throughout the entire clinical study. The risks and potential benefits of participating in the study will be discussed in detail and thoroughly with the subjects and their families. Subjects will be asked to read and review the informed consent form approved by the EC. The investigator will explain the clinical study to the subjects and answer any questions they may have. Subjects will have the opportunity to carefully review the written informed consent form and ask questions before signing it. Subjects will have the opportunity to discuss the study with their representatives and consider it fully before agreeing to participate. Subjects can only begin participating in the study after signing the informed consent form. Subjects may withdraw their consent at any time during the clinical study. A copy of the informed consent

form will be retained by the subject. Even if the consulted patients refuse to participate in this study, their rights will be fully protected; it will be emphasized to them that the quality of their medical care will not be affected in any way.

9.3 Confidentiality of Subject Information

Confidentiality of subject information will be strictly enforced by the investigators, participating research personnel, the sponsor, and its agents. Confidentiality also covers biological samples and genetic testing in addition to the subjects' clinical information. Therefore, the study protocol, documents, data, and all other information generated will be kept strictly confidential.

10. Data Collection and Data Management

10.1 Establishment of EDC Database

The data manager will establish the research data acquisition system and database according to the study protocol and make it available online before the first case is entered. Before use, all EDC users must receive adequate training and obtain corresponding accounts to log in to the system.

10.2 Data Entry and Verification

The investigator or CRC should enter data into the EDC system according to the visit procedures and the eCRF completion guidelines. After submitting the eCRF, the monitor, data manager, and medical personnel should review the data, and any questions during the review should be raised as queries for the investigator or CRC to answer. After the data cleaning is completed, the eCRF needs to be signed and confirmed by the investigator.

10.3 Data Review and Database Lock

After the CRA completes the SDV, the data management personnel and medical personnel will conduct the final quality control of all data in the database, summarize all protocol deviations and violations that occurred during the study, and hold a data review meeting. The principal investigator, co-investigators, statistical experts, and data managers will jointly conduct a data review before statistical analysis. The important content of the review is to determine the analysis dataset to which each case belongs (including FAS, PPS, and SS), the judgment of missing values, and the handling of outliers. Decisions made during the data review cannot be modified, and any decisions must be documented.

The database will then be locked according to the database locking procedure. The locked dataset needs to be properly stored for future reference, and the dataset will be submitted to the statistics department for statistical analysis.

11. Clinical Trial Quality Assurance

To ensure that this trial is conducted strictly in accordance with the clinical research protocol, throughout the entire study, clinical investigators and co-investigators should operate strictly in accordance with the requirements of relevant clinical research regulations, ensuring standardized trial procedures, accurate trial data, and reliable research conclusions.

The investigator is responsible for obtaining the signed Informed Consent Form from each subject or their legal representative. The eCRF should be completed carefully as required, and the monitor will verify its completeness and accuracy and guide the study site staff to make necessary corrections and additions. The laboratory test records, clinical records, and the subjects' original medical records should be retained completely. The clinical monitor will conduct monitoring throughout the trial, regularly visiting each investigator, and ensuring that they can be contacted at any time by phone, fax, or email.

Except for the principal investigator's institution, no one is allowed to modify this protocol. Any necessary changes to the protocol must be made in the form of a protocol amendment, and must be submitted to the EC for approval or filing after obtaining the investigator's signed consent. A detailed description of all modifications should be included in the protocol.

The monitors of this clinical trial have appropriate medical, pharmaceutical, or related professional qualifications and have undergone necessary training. They are familiar with GCP and relevant regulations, monitor and report the progress of the trial and verify the data, ensure that the rights and welfare of the subjects in the clinical trial are protected, and ensure that the data recorded and reported in the trial are accurate, complete, and error-free. They also ensure that the trial adheres to the approved protocol and relevant regulations.

12. References

1. Pelosi, G., et al., *Review article: pulmonary sarcomatoid carcinomas: a practical overview.* Int J Surg Pathol, 2010. **18**(2): p. 103-20.
2. Travis, W.D., et al., *The 2015 World Health Organization Classification of Lung Tumors: Impact of Genetic, Clinical and Radiologic Advances Since the 2004 Classification.* J Thorac Oncol, 2015. **10**(9): p. 1243-1260.
3. Tamaki, T., et al., *Immunohistochemical analysis of NANOG expression and epithelial-mesenchymal transition in pulmonary sarcomatoid carcinoma.* Oncol Lett, 2017. **13**(5): p. 3695-3702.
4. Vieira, T., et al., *Efficacy of first-line chemotherapy in patients with advanced lung sarcomatoid carcinoma.* J Thorac Oncol, 2013. **8**(12): p. 1574-7.
5. Schrock, A.B., et al., *Pulmonary Sarcomatoid Carcinomas Commonly Harbor Either Potentially Targetable Genomic Alterations or High Tumor Mutational Burden as Observed by Comprehensive Genomic Profiling.* J Thorac Oncol, 2017. **12**(6): p. 932-942.
6. Vieira, T., et al., *Sarcomatoid lung carcinomas show high levels of programmed death ligand-1 (PD-L1) and strong immune-cell infiltration by TCD3 cells and macrophages.* Lung Cancer, 2016. **98**: p. 51-58.
7. Kim, S., et al., *Programmed death-1 ligand 1 and 2 are highly expressed in pleomorphic carcinomas of the lung: Comparison of sarcomatous and carcinomatous areas.* Eur J Cancer, 2015. **51**(17): p. 2698-707.
8. Sukrithan, V., et al., *Immune Checkpoint Blockade Is Associated With Durable Responses in Pulmonary Sarcomatoid Carcinoma.* Clin Lung Cancer, 2019. **20**(3): p. e242-e246.
9. Domblides, C., et al., *Efficacy of Immune Checkpoint Inhibitors in Lung Sarcomatoid Carcinoma.* J Thorac Oncol, 2020. **15**(5): p. 860-866.
10. Kaira, K., et al., *Biologic correlates of (1)(8)F-FDG uptake on PET in pulmonary pleomorphic carcinoma.* Lung Cancer, 2011. **71**(2): p. 144-50.
11. Kong, F.W., et al., *First-line albumin-bound paclitaxel/carboplatin plus apatinib in advanced pulmonary sarcomatoid carcinoma: A case series and review of the literature.* Medicine (Baltimore), 2020. **99**(23): p. e20667.
12. Socinski, M.A., et al., *Atezolizumab for First-Line Treatment of Metastatic Nonsquamous NSCLC.* N Engl J Med, 2018. **378**(24): p. 2288-2301.
13. Jin, C. and B. Yang, *Dramatic Response of Pulmonary Sarcomatoid Carcinoma toNivolumab Combined with Anlotinib: A Case Report.* Case Rep Oncol, 2020. **13**(2): p. 601-605.

14. Sun, L., et al., *Pulmonary Sarcomatoid Carcinoma:Experience From SEER Database and Shanghai Pulmonary Hospital*. Ann Thorac Surg, 2020. **110(2)**: p. 406-413.
