# Supplementary material for: Camrelizumab plus famitinib as first-line treatment for locally advanced or metastatic pulmonary sarcomatoid carcinomas (CAPSTONE): a multi-center, single-arm, phase 2 trial
Source: eClinicalMedicine. 2026 Oct 24;101:104285. doi: 10.1016/j.eclinm.2026.104285 (PMC13720253; doi:10.1016/j.eclinm.2026.104285)
Supplement: Supplementary file 2 [file mmc1.docx]

**Supplementary Online Data**

[Supplementary Table 1. CAPSTON recruiting centres	2](#_Toc240306072)

[Supplementary Table 2. Confirmed Tumor Response in the EAS	3](#_Toc240306073)

[Supplementary Table 3. Subsequent anticancer therapies after disease progression	4](#_Toc240306074)

[Supplementary Table 4. Quality of Life scores in EORTC QLQ-C30 questionnaire	5](#_Toc240306075)

[Supplementary Table 5. Treatment-related adverse events leading to dose reductions of famitinib	6](#_Toc240306076)

[Supplementary Table 6. Immune-related adverse events	7](#_Toc240306077)

[Supplementary Table 7. Serious treatment-related adverse events	8](#_Toc240306078)

[Supplementary Table 8. Types of EGFR alterations in 19% of the patients with available genomic data	9](#_Toc240306079)

[Supplementary Table 9. The clinical information of seven patients who underwent single-cell sequencing	10](#_Toc240306080)

[Supplementary Figure 1. Forest plot of subgroup analysis for objective response rate.	11](#_Toc240306081)

[Supplementary Figure 2. Tumor response.	12](#_Toc240306082)

[Supplementary Figure 3. Kaplan-Meier estimates for progression-free survival in patients with spindle cell carcinoma or other histological types.	13](#_Toc240306083)

[Supplementary Figure 4. Kaplan-Meier estimates for progression-free survival (A) and overall survival (B) according to PD-L1 status.	14](#_Toc240306084)

[Supplementary Figure 5. Kaplan-Meier estimates for progression-free survival (A) and overall survival (B) in EAS.	15](#_Toc240306085)

[Supplementary Figure 6. Mean change from baseline in EORTC QLQ-C30 for global quality of life.	16](#_Toc240306086)

[Supplementary Figure 7. The landscape of genomic alterations in patients with pulmonary sarcomatoid carcinoma.	17](#_Toc240306087)

[Supplementary Figure 8. scRNA-seq overview and cellular composition differences in patients with different treatment responses.	18](#_Toc240306088)

[Supplementary Figure 9. T-cell functional differences in patients with different treatment responses.	19](#_Toc240306089)

[Supplementary Figure 10. Correlation analysis of CD8^+^ T-cell and CD4^+^ T-cell subpopulation proportions with the best change in target lesions from baseline in patients.	20](#_Toc240306090)

[Supplementary Figure 11. T-cell differential gene expression and immune cell-cell communication.	21](#_Toc240306091)

**Supplementary Table 1. CAPSTON recruiting centres**

| Sites | Principal Investigator | Number of patients screened | Number of patients enrolled |
| --- | --- | --- | --- |
| Affiliated Tongji Hospital of Tongji Medical College | Qian Chu | 21 | 16 |
| Hubei Cancer Hospital | Bin Yang | 4 | 3 |
| The Second Xiangya Hospital of Central South University | Xianling Liu | 3 | 3 |
| Henan Provincial People’s Hospital | Shundong Cang | 3 | 2 |
| Henan Cancer Hospital | Huijuan Wang | 2 | 2 |
| Renmin Hospital of Wuhan University | Qibin Song | 2 | 2 |
| The First Affiliated Hospital of Xiamen University | Feng Ye | 0 | 0 |
| The First Affiliated Hospital of Shandong First Medical University | Jun Wang | 0 | 0 |

**Supplementary Table 2. Confirmed Tumor Response in the EAS**

|  | **Patients (*n* = 27)** |
| --- | --- |
| Confirmed best overall response |  |
| Complete response | 0 |
| Partial response | 12 (44.4) |
| Stable disease | 12 (44.4) |
| Progressive disease | 3 (11.1) |
| Not evaluable | 0 |
| Objective response rate | 12 (44.4%, 25.5-64.7) |
| Disease control rate | 24 (88.9%, 70.8-97.7) |

Data are *n* (%) or *n* (%, 95% CI). CI, confidence interval; EAS, efficacy analysis set.

**Supplementary Table 3. Subsequent anticancer therapies after disease progression**

|  | **Patients (*n* = 20)** |
| --- | --- |
| **Received subsequent anticancer therapy** | 10 (50.0) |
| PD-1 inhibitors plus chemotherapy | 2 (10.0) |
| PD-1 inhibitors plus chemotherapy and antiangiogenic agents | 2 (10.0) |
| PD-L1 inhibitor monotherapy | 1 (5.0) |
| PD-L1 inhibitor plus antiangiogenic agent | 1 (5.0) |
| Specific regimen unavailable* | 4 (20.0) |
| **No subsequent anticancer therapy** | 6 (30.0) |
| **Subsequent treatment status unknown^#^** | 4 (20.0) |

Data are presented as *n* (%). *Patients were confirmed to have received subsequent anticancer therapy, but information on the specific treatment regimen was unavailable. ^#^Follow-up information regarding subsequent anticancer therapy could not be obtained; therefore, it could not be determined whether these patients received subsequent anticancer therapy. PD-1, programmed cell death-1; PD-L1, programmed cell death ligand-1.

**Supplementary Table 4. Quality of Life scores in EORTC QLQ-C30 questionnaire**

| **Subscale** | **Baseline** | | **Cycle 5** | | **Cycle 9** | | **Cycle 13** | | **Cycle 17** | | |
| --- | --- | --- | --- | --- | --- | --- | --- | --- | --- | --- | --- |
|  | **N** | **Mean (95% Cl)** | **N** | **Mean (95% Cl)** | **N** | **Mean (95% Cl)** | **N** | **Mean (95% Cl)** | **N** | **Mean (95% Cl)** |  |
| **Global health status/QoL** | 26 | 61.9 (51.0, 72.8) | 17 | 65.7 (56.0, 75.4) | 11 | 69.7 (58.4, 81.0) | 10 | 70.8 (55.6, 86.0) | 7 | 78.6 (59.2, 97.9) |  |
| **Functional scales** |  |  |  |  |  |  |  |  |  |  |  |
| Physical functioning | 26 | 79.5 (71.9, 87.1) | 17 | 80.0 (72.1, 87.9) | 11 | 83.0 (69.4, 96.7) | 10 | 78.0 (62.9, 93.1) | 7 | 81.9 (68.7, 95.1) |  |
| Role functioning | 26 | 76.3 (66.9, 85.6) | 17 | 71.6 (58.4, 84.7) | 11 | 74.2 (60.7, 87.8) | 10 | 71.7 (51.4, 92.0) | 7 | 78.6 (55.5, 101.6) |  |
| Emotional functioning | 26 | 80.1 (72.9, 87.4) | 17 | 80.4 (70.7, 90.1) | 11 | 83.3 (72.4, 94.2) | 10 | 80.8 (69.2, 92.4) | 7 | 88.1 (74.1, 102.1) |  |
| Cognitive functioning | 26 | 89.7 (82.9, 96.6) | 17 | 85.3 (77.9, 92.6) | 11 | 86.4 (75.4, 97.4) | 10 | 81.7 (68.5, 94.8) | 7 | 88.1 (68.8, 107.4) |  |
| Social functioning | 26 | 75.6 (67.5, 83.8) | 17 | 73.5 (63.5, 83.6) | 11 | 68.2 (53.6, 82.7) | 10 | 71.7 (60.4, 83.0) | 7 | 71.4 (43.7, 99.2) |  |
| **Symptom scales** |  |  |  |  |  |  |  |  |  |  |  |
| Fatigue | 26 | 29.5 (22.0, 37.0) | 17 | 22.9 (13.5, 32.2) | 11 | 21.2 (8.5, 33.9) | 10 | 27.8 (9.7, 45.8) | 7 | 20.6 (5.6, 35.7) |  |
| Nausea and vomiting | 26 | 9.6 (1.5, 17.7) | 17 | 4.9 (-0.1, 9.9) | 11 | 4.5 (-2.7, 11.8) | 10 | 5.0 (-0.8, 10.8) | 7 | 7.1 (-5.0, 19.3) |  |
| Pain | 26 | 21.2 (12.3, 30.0) | 17 | 24.5 (10.6, 38.4) | 11 | 18.2 (7.6, 28.8) | 10 | 16.7 (4.1, 29.2) | 7 | 9.5 (-2.6, 21.7) |  |
| Dyspnea | 26 | 26.9 (15.5, 38.4) | 17 | 27.5 (13.6, 41.3) | 11 | 24.2 (4.0, 44.5) | 10 | 20.0 (3.3, 36.7) | 7 | 19.0 (-15.9, 54.0) |  |
| Insomnia | 26 | 25.6 (15.3, 35.9) | 17 | 27.5 (13.6, 41.3) | 11 | 12.1 (0.8, 23.4) | 10 | 16.7 (4.1, 29.2) | 7 | 9.5 (-5.5, 24.6) |  |
| Appetite loss | 26 | 21.8 (12.5, 31.1) | 17 | 19.6 (7.4, 31.8) | 11 | 15.2 (3.5, 26.8) | 10 | 23.3 (7.2, 39.4) | 7 | 23.8 (0.5, 47.1) |  |
| Constipation | 26 | 19.2 (9.8, 28.7) | 17 | 11.8 (-1.7, 25.2) | 11 | 24.2 (1.6, 46.8) | 10 | 10.0 (-1.5, 21.5) | 7 | 4.8 (-6.9, 16.4) |  |
| Diarrhea | 26 | 5.1 (-1.1, 11.4) | 17 | 11.8 (3.3, 20.2) | 11 | 18.2 (2.8, 33.6) | 10 | 30.0 (6.3, 53.7) | 7 | 28.6 (0.8, 56.3) |  |
| Financial difficulties | 26 | 38.5 (25.4, 51.5) | 17 | 45.1 (29.1, 61.1) | 10 | 43.3 (20.7, 66.0) | 10 | 43.3 (27.2, 59.4) | 7 | 38.1 (5.1, 71.1) |  |

**Supplementary Table 5. Treatment-related adverse events leading to dose reductions of famitinib**

|  | Patients (*n* = 28) | |
| --- | --- | --- |
|  | Any grade | Grade ≥ 3 |
| Any | 4 (14.3) | 0 |
| White blood cell count decreased | 2 (7.1) | 0 |
| Nausea | 1 (3.6) | 0 |
| Fever | 1 (3.6) | 0 |
| Oral mucositis | 1 (3.6) | 0 |
| Lymphocyte count decreased | 1 (3.6) | 0 |
| Decreased appetite | 1 (3.6) | 0 |
| Headache | 1 (3.6) | 0 |
| Hand-foot syndrome | 1 (3.6) | 0 |
| Neutrophil count decreased | 1 (3.6) | 0 |

Data are *n* (%).

**Supplementary Table 6. Immune-related adverse events**

| Immune-related adverse events | Patients (*n* = 28) | |
| --- | --- | --- |
|  | Any grade | Grade ≥ 3 |
| Any | 11 (39.3) | 1 (3.6) |
| Aspartate aminotransferase increased | 5 (17.9) | 1 (3.6) |
| Alanine aminotransferase increased | 4 (14.3) | 0 |
| Hypothyroidism | 4 (14.3) | 0 |
| Reactive cutaneous capillary endothelial proliferation | 3 (10.7) | 0 |
| Adrenal insufficiency | 2 (7.1) | 0 |
| Arrhythmia | 1 (3.6) | 0 |
| Infectious pneumonia | 1 (3.6) | 0 |
| Hyperthyroidism | 1 (3.6) | 0 |
| Pruritus | 1 (3.6) | 0 |
| Rash | 1 (3.6) | 0 |
| Conjugated bilirubin increased | 1 (3.6) | 0 |
| Pulmonary inflammation | 1 (3.6) | 0 |
| Diarrhoea | 1 (3.6) | 0 |
| Alkaline phosphatase increased | 1 (3.6) | 0 |
| Blood bilirubin increased | 1 (3.6) | 0 |
| Blood creatinine increased | 1 (3.6) | 0 |

Data are *n* (%).

**Supplementary Table 7. Serious treatment-related adverse events**

|  | Patients (*n* = 28) | |
| --- | --- | --- |
|  | Any grade | Grade ≥ 3 |
| Any | 6 (21.4) | 4 (14.3) |
| Alanine aminotransferase increased | 2 (7.1) | 1 (3.6) |
| Aspartate aminotransferase increased | 2 (7.1) | 1 (3.6) |
| Platelet count decreased | 2 (7.1) | 2 (7.1) |
| Unknown death | 2 (7.1) | 2 (7.1) |
| Haemoptysis | 1 (3.6) | 1 (3.6) |
| Hand-foot syndrome | 1 (3.6) | 0 |
| Decreased appetite | 1 (3.6) | 0 |

Data are *n* (%).

**Supplementary Table 8. Types of EGFR alterations in 19% of the patients with available genomic data**

| Patient number | EGFR alteration | Exon/region | Alteration type | EGFR-sensitizing mutation |
| --- | --- | --- | --- | --- |
| 1015 | p.D1008N (c.3022G>A) | Exon 25 | Missense variant | No |
| 7001 | Amplification | EGFR locus | Amplification | No |
| 5002 | p.H888R (c.2663A>G) | Exon 22 | Missense variant | No |
| 11001 | Amplification | EGFR locus | Amplification | No |
| 11002 | Amplification | EGFR locus | Amplification | No |

**Supplementary Table 9. The clinical information of seven patients who underwent single-cell sequencing**

| **Sample ID** | **Gender** | **Age at biopsy (years)** | **Smoking History** | **Biopsy Site** | **Tissue** | **Primary/Metastatic** | **Stage** | **Progression-free survival (months)** | **Best change in target lesions from baseline (%)** |
| --- | --- | --- | --- | --- | --- | --- | --- | --- | --- |
| 01004 | Male | 65 | Former | Lung | Tumor | Primary | IVA | 1.6 | -11.02 |
| 01005 | Male | 63 | Former | Lung | Tumor | Primary | IVA | 4.3 | -27.36 |
| 01007 | Male | 59 | Never | Lung | Tumor | Primary | IVB | 7.8 | -35.54 |
| 01001 | Male | 45 | Never | Lung | Tumor | Primary | IVA | 10.4 | -53.97 |
| 01012 | Male | 64 | Never | Lung | Tumor | Primary | IV | 17.5 | -65.32 |
| 01010 | Male | 59 | Former | Lung | Tumor | Primary | IV | 9.7 | -66.79 |
| 01018 | Male | 59 | Never | Lung | Tumor | Primary | IIIB | 12.6 | -74.33 |

**Supplementary Figure 1. Forest plot of subgroup analysis for objective response rate.**

CI, confidence interval; ORR, objective response rate; PD-L1, programmed cell death ligand-1; TMB, tumor mutational burden.

**Supplementary Figure 2. Tumor response.**

(**A**) Kaplan-Meier estimates for duration of response in patients with a confirmed partial response. (**B**) Waterfall plot for best change in target lesions from baseline, with color of bar indicating confirmed best response. (**C**) Swimming plot for treatment exposure in all patients enrolled.

CI, confidence interval; PD-L1, programmed cell death ligand 1.

**Supplementary Figure 3.** **Kaplan-Meier estimates for progression-free survival in patients with spindle cell carcinoma or other histological types.**

CI, confidence interval.

**Supplementary Figure 4.** **Kaplan-Meier estimates for progression-free survival (A) and overall survival (B) according to PD-L1 status.**

CI, confidence interval; NE, not estimable; PD-L1, programmed cell death ligand-1.

**Supplementary Figure 5.** **Kaplan-Meier estimates for progression-free survival (A) and overall survival (B) in EAS.**

CI, confidence interval; EAS, efficacy analysis set; NE, not estimable.

**Supplementary Figure 6.** **Mean change from baseline in EORTC QLQ-C30 for global quality of life.**

C, cycle; SE, standard error.

**Supplementary Figure 7. The landscape of genomic alterations in patients with pulmonary sarcomatoid carcinoma.**

(A) Landscape of genomic alterations in baseline tumor samples. (B) Kaplan-Meier curves for PFS and OS according to TMB and TNB. PFS, progression-free survival; OS, overall survival; TMB, tumor mutational burden; TNB, tumor neoantigen burden.

**Supplementary Figure 8.** **scRNA-seq overview and cellular composition differences in patients with different treatment responses.**

(A) Schematic diagram of the scRNA-seq process with BioRender. (B) UMAP plots of cancer tissue cells, stratified by patients’ mPFS. (C) UMAP plots of cancer tissue cells, stratified by patients. (D) Scale plot of cancer tissue cells, stratified by patients’ mPFS. (E) The circular plot shows the immune cell composition in different mPFS groups. mPFS, median progression-free survival; PSC, pulmonary sarcomatoid carcinoma.

**Supplementary Figure 9.** **T-cell functional differences in patients with different treatment responses.**

(A) UMAP plot highlighting the major subclusters of T cells, color-coded by cell type. (B) Windrose plot displaying the proportion of major T-cell subpopulations and NK cell subpopulations, stratified by patients’ mPFS. (C) Violin plot displaying CD8^+^ T-cell functional scores in patients with different treatment responses. (D) Dot plot displaying the expression of PDCD1 (i.e. PD-1), IFNG, and MKI67 in CD8^+^ T cells from patients with different treatment responses. mPFS, median progression-free survival.

**Supplementary Figure 10.** **Correlation analysis of CD8^+^ T-cell and CD4^+^ T-cell subpopulation proportions with the best change in target lesions from baseline in patients.**

(A) Correlation analysis assessing the relationship between the best change in target lesions from baseline and the proportion of the CD8^+^ T cell subgroup (Spearman correlation). (B) Correlation analysis assessing the relationship between the best change in target lesions from baseline and the proportion of the CD4^+^ T-cell subgroup (Spearman correlation). Numbers represent different cell clusters.

**Supplementary Figure 11.** **T-cell differential gene expression and immune cell-cell communication.**

(A) Volcano plots showing the differential gene expression between CD8-C0 and other CD8^+^ T-cell subpopulations, and between CD4-C0 and other CD4^+^ T-cell subpopulations (red: upregulated genes; blue: downregulated genes; grey: non-significant genes). (B) MultiNicheNet analysis of intercellular communication among immune subpopulations, stratified by patients’ mPFS. mPFS, median progression-free survival.
